# Substrate specificities and reaction kinetics of the yeast oligosaccharyltransferase isoforms

**DOI:** 10.1016/j.jbc.2021.100809

**Published:** 2021-05-21

**Authors:** Jillianne Eyring, Chia-Wei Lin, Elsy Mankah Ngwa, Jérémy Boilevin, Giorgio Pesciullesi, Kaspar P. Locher, Tamis Darbre, Jean-Louis Reymond, Markus Aebi

**Affiliations:** 1 Institute of Microbiology, Department of Biology, ETH Zürich, Zürich, Switzerland; 2 Functional Genomics Center Zürich, University of Zürich/ETH Zürich, Zürich, Switzerland; 3 Department of Chemistry and Biochemistry, University of Berne, Bern, Switzerland; 4 Institute of Molecular Biology and Biophysics, Department of Biology, ETH Zürich, Zürich, Switzerland

**Keywords:** oligosaccharyltransferase, OST, carbohydrate structure, enzyme kinetics, glycoprotein biosynthesis, glycosylation, glycosylation inhibitor, membrane enzyme, protein complex, yeast, ACN, acetonitrile, CHS, cholesteryl hemisuccinate, DDM, *n*-dodecyl-β-D-maltopyranoside, ER, endoplasmic reticulum, FA, formic acid, FDR, false discovery rate, HCD, higher-energy collisional dissociation, LLO, lipid-linked oligosaccharide, OST, oligosaccharyltransferase, TAMRA, tetramethylrhodamine, XIC, extracted ion chromatography

## Abstract

Oligosaccharyltransferase (OST) catalyzes the central step in N-linked protein glycosylation, the transfer of a preassembled oligosaccharide from its lipid carrier onto asparagine residues of secretory proteins. The prototypic hetero-octameric OST complex from the yeast *Saccharomyces cerevisiae* exists as two isoforms that contain either Ost3p or Ost6p, both noncatalytic subunits. These two OST complexes have different protein substrate specificities *in vivo*. However, their detailed biochemical mechanisms and the basis for their different specificities are not clear. The two OST complexes were purified from genetically engineered strains expressing only one isoform. The kinetic properties and substrate specificities were characterized using a quantitative *in vitro* glycosylation assay with short peptides and different synthetic lipid-linked oligosaccharide (LLO) substrates. We found that the peptide sequence close to the glycosylation sequon affected peptide affinity and turnover rate. The length of the lipid moiety affected LLO affinity, while the lipid double-bond stereochemistry had a greater influence on LLO turnover rates. The two OST complexes had similar affinities for both the peptide and LLO substrates but showed significantly different turnover rates. These data provide the basis for a functional analysis of the Ost3p and Ost6p subunits.

Asparagine-linked glycosylation (*N*-glycosylation) of proteins is one of the most common covalent posttranslational protein modifications in eukaryotes. Homologous processes are found in archaea and bacteria (1). The N-linked glycans fulfill a multitude of functions, such as regulating and controlling protein folding and intracellular trafficking or defining interactions at the cell surface (2, 3, 4). In a key step of this pathway, the oligosaccharyltransferase (OST) enzyme transfers a preassembled oligosaccharide from a lipid carrier onto an asparagine residue on proteins in the endoplasmic reticulum (ER) (5). The modified asparagine residue is part of the consensus sequon N-X-(S/T). OST binds both the acceptor protein and the donor lipid-linked oligosaccharide (LLO) and catalyzes the formation of a glycosidic bond between the amide nitrogen of the asparagine side chain and the C1 carbon of the reducing end *N*-acetylglucosamine (GlcNAc) residue of the oligosaccharide. High-resolution structures and biochemical studies on the bacterial OST, PglB from *Campylobacter lari*, propose mechanisms for peptide and LLO binding, amide activation, and catalysis (6, 7, 8, 9, 10, 11).

In animals, plants, and fungi, OST is a multi-subunit protein complex in which the STT3 protein is the conserved catalytic subunit containing the active site. Stt3p is homologous to the single-subunit OST enzymes found in some bacteria (*e.g.*, PglB from *C. lari*), archaea (*e.g.*, AglB from *Pyrococcus furiosus*), and eukaryotic kinetoplastids (*e.g.*, STT3 from *Leishmania major* and *Trypanosoma brucei*) (12). Many residues essential for OST function are highly conserved, and the superposition of the substrate-bound structures of PglB to the structure of the yeast Stt3p reveals that the active sites of Stt3 and PglB are very similar (13, 14). The key catalytic residues that bind the peptide and LLO substrates in the substrate-bound PglB structures are conserved in the yeast Stt3p and located at the same positions with respect to both substrates and the coordinating metal ion (6, 10).

Despite the same reaction mechanism, major differences in the substrate specificities of OSTs from different organisms are reported. In most eukaryotes, the LLO substrate is large and contains the GlcNAc_2_Man_9_Glc_3_ oligosaccharide, but smaller glycan substrates exist (1, 15, 16). It also appears that the transferred oligosaccharides are shorter in prokaryotic protein glycosylation. In terms of the polypeptide substrate, the N-X-(S/T) sequon requirement remains the same for all OSTs, yet animals, plants, and fungi have a wider range of protein substrates and a larger number of glycosylation sites modified in their proteomes (1, 17, 18), compared with bacteria, archaea, and unicellular eukaryotic kinetoplastids (19, 20, 21). This increase in glycosylated sites correlates with an increase in OST complexity through the acquisition of additional subunits. It is thought that the additional noncatalytic subunits enhance glycosylation efficiency of the catalytic STT3 protein by facilitating protein and LLO substrate binding (1, 12).

The prototypic OST complex from the budding yeast *Saccharomyces cerevisiae* is composed of the eight subunits Ost1p, Ost2p, Ost4p, Ost5p, Stt3p, Swp1p, Wbp1p, and either Ost3p or Ost6p (12). Ost3p and Ost6p are nonessential homologous oxidoreductases that assemble as the last subunit into separate OST complexes, resulting in two isoforms of yeast OST that coexist (22, 23, 24). Multicellular animals and plants have an additional layer of complexity in that they express two different OST complexes that contain one of the two Stt3 paralogs, Stt3A and Stt3B (25, 26, 27). The Stt3A complex associates directly with the translocon *via* the subunit DC2 and performs cotranslational glycosylation, whereas the Stt3B complex incorporates an oxidoreductase subunit instead of DC2 and performs posttranslocational glycosylation (13, 28, 29, 30, 31). In the yeast OST, an Stt3B-type OST, the oxidoreductases Ost3p or Ost6p are incorporated, equivalent to the mutually exclusive incorporation of the TUSC3 and MagT1 oxidoreductase subunits in mammalian Stt3B complexes (31, 32). Interestingly, while nearly all organisms that express multi-subunit OSTs encode an oxidoreductase subunit (OST3 homolog) (12), two functional homologs of oxidoreductases appear to be present in all vertebrates and some fungi, suggesting an important function of such redundancy (33).

Ost3p and Ost6p are both oxidoreductases that can interact with the polypeptide substrate and are thought to slow down the oxidative folding of the glycoprotein substrate to transiently improve accessibility of available glycosylation sequons and increase glycosylation efficiency (34, 35). Indeed, the high-resolution structures of the yeast and the mammalian Stt3B OST show that this subunit not only directly interacts with the catalytic subunit, Stt3p, but its thioredoxin domain is also positioned right across from the Stt3p active site (13, 14, 31). In yeast, Ost3p- and Ost6p-containing complexes have different peptide substrate preferences *in vivo*, and the Ost3p-containing complex (OST3 complex) is required for efficient glycosylation of a larger subset of proteins than the Ost6p-containing complex (OST6 complex) (34). Furthermore, the OST3 complex is more abundant in yeast (33, 36, 37) and has a higher relative enzymatic activity than the OST6 complex *in vitro* (38, 39).

To understand the differences between the OST3 and OST6 complexes, we characterized the substrate specificities and enzyme kinetics of the two OST isoforms *in vitro*. We found that the two complexes had similar affinities to short, synthetic peptide and LLO substrates but differed significantly in their catalytic efficiency.

## Results

### Isolation and characterization of the two yeast OST complex isoforms

**Figure 1:**
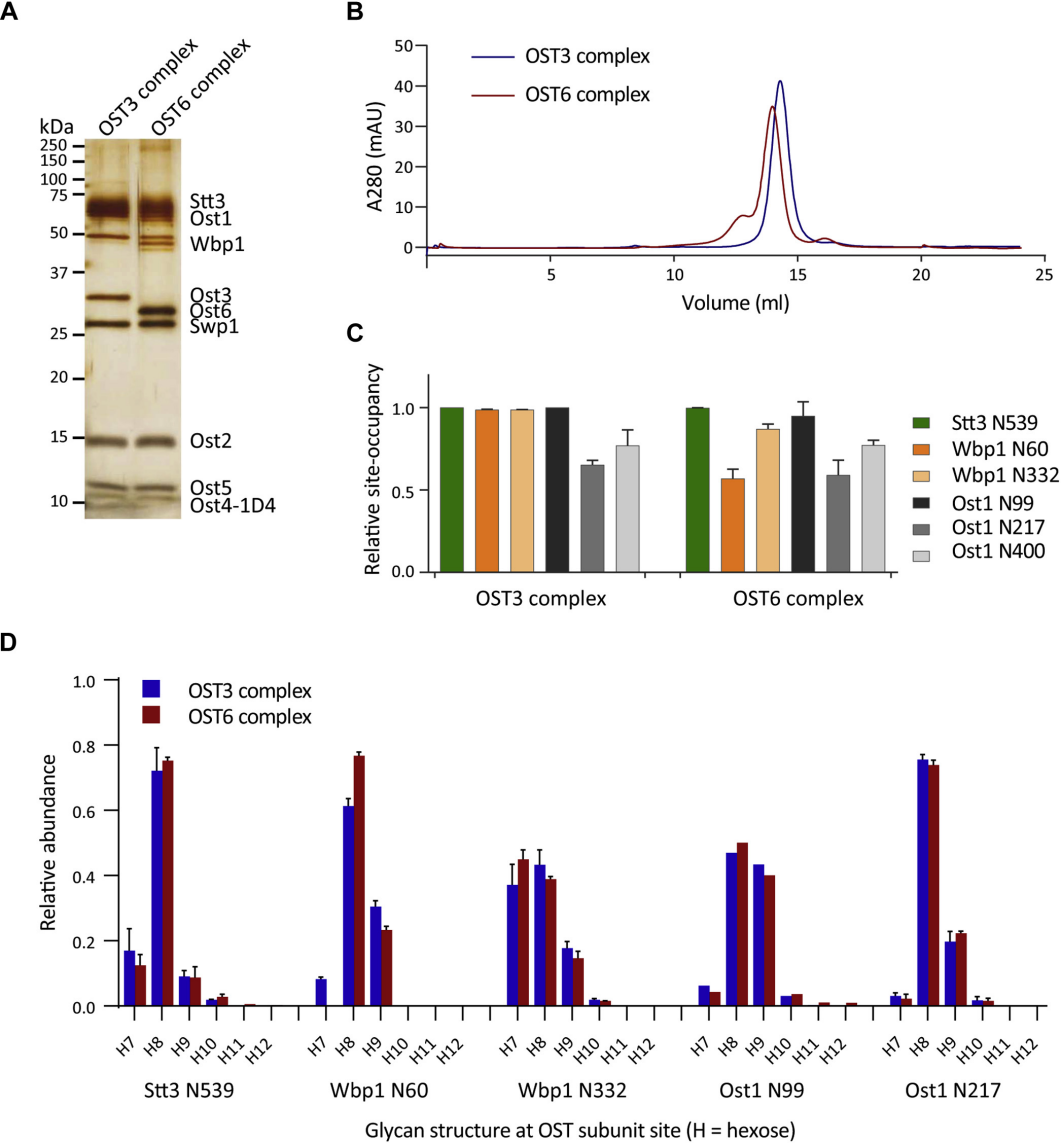
**Characterization of the purified yeast OST complex isoforms and analysis of glycosylation site occupancy and glycan structures on the OST glycoproteins of each complex.***A*, SDS-PAGE (14% acrylamide) silver staining of 0.2 μg purified OST complexes containing either Ost3p (OST3 complex) or Ost6p (OST6 complex). Multiple bands of Ost1p and Wbp1p represent the fully and hypoglycosylated forms of the proteins. Ost4p is tagged with the 1D4 epitope for purification. *B*, overlaid size-exclusion chromatography profiles on a Superose 6 Increase 10/300 Gl column (GE Healthcare) of the fully purified OST3 complex (blue) and OST6 complex (*red*) after a previous size-exclusion chromatography step. The large peaks coelute around 14 ml, corresponding to purified complexes of a similar size (∼280 kDa). *C*, glycosylation site occupancy of glycoproteins Stt3p, Wbp1p, and Ost1p of the OST complexes. Purified OST3 and OST6 complexes were prepared for mass spectrometry, digested by trypsin, and treated with EndoH. The glycopeptides and nonglycosylated peptides were detected by LC-MS/MS and the relative amount of glycopeptide to nonglycosylated peptide is reported as relative occupancy of the glycosylation site. Error bars indicate standard deviations from the mean of three independent protein purifications (n = 3). The XICs and MS/MS spectra of identified peptides are shown in Figure S1. *D*, the relative abundance of glycan structures at different sites of the glycoproteins Stt3p, Wbp1p, and Ost1p in the OST3 and OST6 complexes. Purified OST3 and OST6 complexes were prepared for mass spectrometry, digested by trypsin, and glycopeptides were detected by LC-MS/MS. H7, H8, H9, H10, H11, H12 represent the number of hexoses (mannose or glucose) on the glycan in addition to the two core GlcNAcs. Error bars indicate the standard deviation from the mean of three independent protein purifications (n = 3), except for Ost1 N99 (n = 1). The XICs and MS/MS spectra of identified peptides are shown in Figure S2.

The two isoforms of the yeast OST complex that contain either of the functional homologs, Ost3p or Ost6p, were purified separately from strains expressing only one type of complex by deleting either *OST6* or *OST3* and overexpressing the desired subunit (*OST3* or *OST6*, respectively) to ensure the assembly of complete complexes in the cell (22, 23). Insertion of a 1D4 epitope tag at the C-terminus of the Ost4p subunit in these strain backgrounds allowed for a very efficient purification of complete OST complexes of a single isoform, as shown previously for determining the structure of the yeast OST (13). Silver staining of purified OST complexes showed that all eight subunits were present and either Ost3p or Ost6p was solely incorporated into each respective complex (Fig. 1*A*). Both complexes also ran as single monodispersed complexes in size-exclusion chromatography and had a very similar size (Fig. 1*B*).

Aside from the presence of either Ost3p or Ost6p, the only other difference observed between the purified OST isoforms was that the OST6 complex contained hypoglycosylated subunits (Fig. 1*A*). Analysis of the glycosylation site occupancy of the OST complex glycoproteins Stt3p, Wbp1p, and Ost1p by mass spectrometry confirmed that the OST6 complex was generally less efficiently glycosylated than the OST3 complex, but the Stt3p N539 glycosite was fully glycosylated in both OST complexes (Fig. 1*C*). Both Wbp1p glycosylation sites were fully modified in the OST3 complex, but their site occupancy was reduced in the OST6 complex, particularly that of Wbp1p N60 (reduced by 50%). Regarding the Ost1p glycosylation sites, only Ost1p N99 was fully glycosylated in the OST3 complex and the occupancies of Ost1p N99 and N217 were only slightly reduced in the OST6 complex, but not to the same extent as that observed for the Wbp1p glycosites.

The glycan structures at each glycosylation site were determined by mass spectrometry. We found glycan heterogeneity at each site analyzed, but the site-specific glycan structure profile was very similar in both OST3 and OST6 complexes (Fig. 1*D*). In agreement with our understanding of the yeast glycan modification pathway in the ER and Golgi (40), the most abundant glycan structure on Stt3p N539, Wbp1p N60, and Ost1p N217 was identified as Man_8_GlcNAc_2_, confirming a previous report on Stt3p N539 (41). The glycan profile of Wbp1p N332 showed that it carried either Man_8_GlcNAc_2_ or Man_7_GlcNAc_2_ structures. Ost1p N99 carried either Man_8_GlcNAc_2_ or Man_9_GlcNAc_2_ structures, but we also detected some larger glycans with up to ten hexose units (Fig. 1*D*).

### *In vitro* assay for yeast OST activity

**Figure 2:**
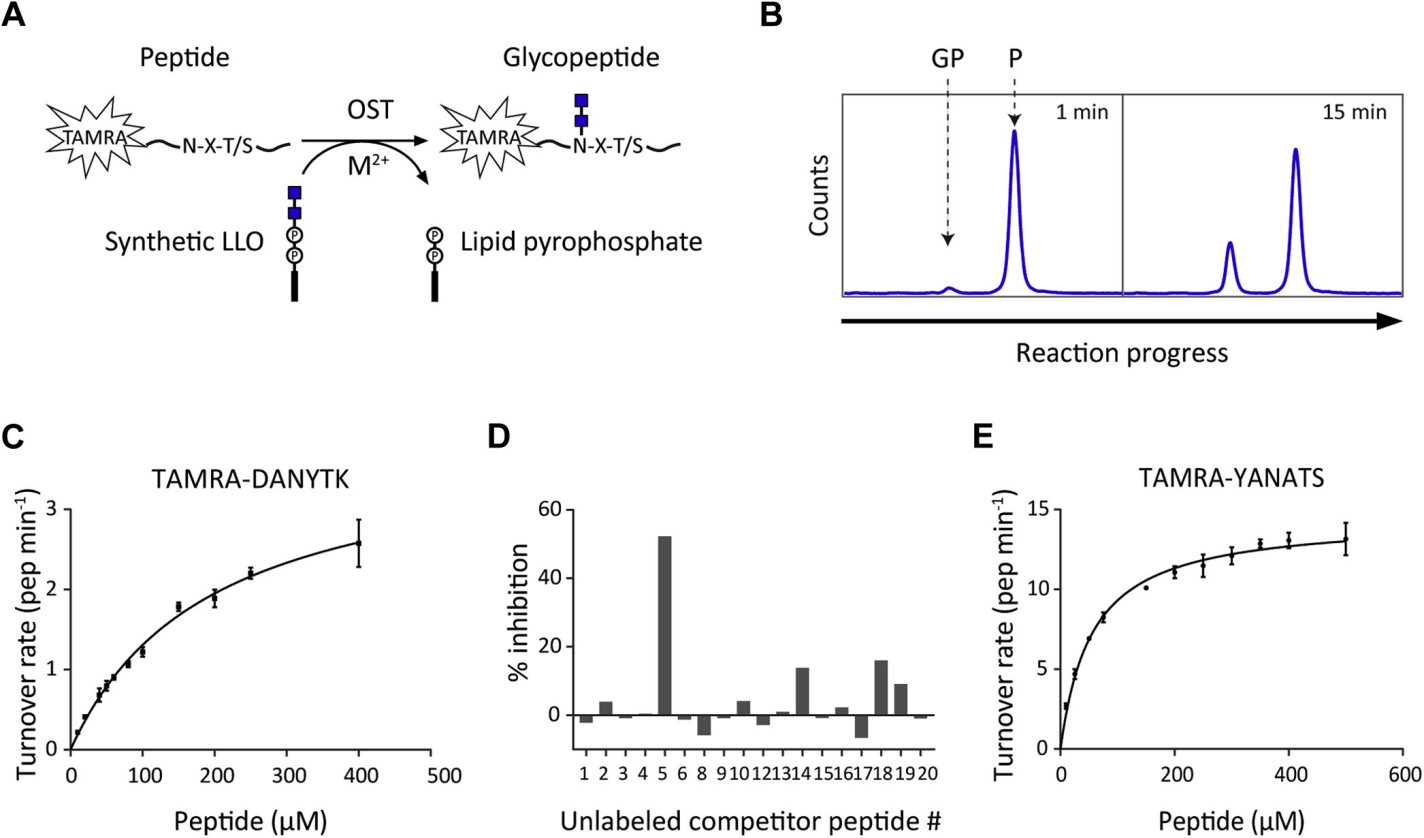
***In vitro* glycosylation assay for OST activity and peptide sequence preferences.***A*, schematic representation of the *in vitro* reaction setup for yeast OST activity. The peptide substrate containing the glycosylation sequon N-X-(S/T) is labeled with the TAMRA fluorophore at the N-terminus. Incubation with OST, synthetic LLO, and a divalent metal ion (Mn^2+^preferred) results in the transfer of the sugar moiety (two GlcNAcs, blue squares) from the LLO to the fluorescently labeled peptide, yielding a glycopeptide product. *B*, measurement and analysis of the *in vitro* assay shown in *A*. Fluorescently labeled peptide (P) substrate and glycopeptide (GP) product are separated by reverse-phase chromatography using UPLC. The glycopeptide elutes earlier than the peptide and amounts of glycopeptide are quantified at different reaction time points by peak integration. *C*, quantification of kinetic parameters for synthetic peptide TAMRA-DANYTK. Reactions were performed with 2.8 μg purified extract containing OST3 complex before size-exclusion chromatography (approximately 1 μM OST3 complex), 150 μM LLO C20, and varying concentrations of the peptide. Glycopeptide product was measured by UPLC and turnover rates were determined by linear regression of the initial phase of the reaction and taking into account the enzyme concentration. The mean of three reactions is shown and the data were fitted by nonlinear regression according to the Michaelis–Menten formula (R^2^ = 0.9803) using Prism. Error bars indicate standard deviations from the mean (n = 3). The derived kinetic parameters are summarized in Table 1. *D*, screening glycosylatable peptides derived from highly glycosylated yeast sequences. Reactions were performed with 0.4 μM purified OST3 complex, 150 μM LLO C20, 25 μM TAMRA-DANYTK, and 25 μM unlabeled competitor peptide. TAMRA-DANYTK glycopeptide product formed after 30 min was measured by UPLC and % inhibition was determined relative to a control reaction containing no unlabeled competitor peptide. *E*, quantification of kinetic parameters for synthetic peptide TAMRA-YANATS. Reactions were performed with 0.1 μM purified OST3 complex, 100 μM LLO C20, and varying concentrations of the peptide. Turnover rates were determined as in *C*. The mean of three reactions is shown and the data were fitted by nonlinear regression according to the Michaelis–Menten formula (R^2^ = 0.9794) using Prism. Error bars indicate standard deviations from the mean (n = 3). The derived kinetic parameters are summarized in Table 1. P, phosphate; TAMRA, tetramethylrhodamine.

OST activity was determined using a short peptide labeled with the TAMRA (tetramethylrhodamine) fluorophore and synthetic LLO analogs with two GlcNAc residues (chitobiose) as the sugar moiety, as used previously (13, 42). Incubation of these synthetic substrates with purified yeast OST resulted in the formation of a fluorophore-labeled glycopeptide product (Fig. 2*A*). We detected and quantified the formation of glycopeptide product using reverse phase UPLC (ultra performance liquid chromatography) (43) instead of a gel-based visualization of glycopeptide formation (Fig. 2*B*). The peak corresponding to the earlier-eluting glycopeptide increased with reaction time, and peak integration allowed an accurate and sensitive quantification of product formation.

### OST3 complex displays preferences for peptide substrates

A quantitative analysis of the peptide substrate specificity of the OST3 complex *in vitro* was first performed using the fluorescently labeled hexapeptide TAMRA-DANYTK-NH_2_ as the acceptor peptide (44). The initial reaction velocity was measured at various peptide concentrations and a fixed synthetic LLO concentration (Fig. 2*C*). The data were fitted according to Michaelis–Menten kinetics and apparent K_M_ and k_cat_ values were derived (Table 1). Saturation of the peptide substrate could not be reached, but the high apparent K_M_ of around 200 μM indicated that the short peptide used was a rather poor substrate. To obtain a more optimal peptide substrate for the yeast OST, we screened a library of peptide sequences containing a glycosylation sequon derived from a list of efficiently glycosylated yeast proteins *in vivo* (37) (Table S1). The screen was performed by adding unlabeled synthetic peptides as a competitor substrate to *in vitro* reactions containing the fluorescently labeled TAMRA-DANYTK. Some peptides slightly inhibited the turnover of the labeled TAMRA-DANYTK peptide, while Peptide 5 (ADTYANATSDVL) inhibited the reaction by 50% (Fig. 2*D*). To directly show that this peptide sequence was indeed a better acceptor substrate than TAMRA-DANYTK, we measured the initial turnover of TAMRA-labeled Peptide 5 (TAMRA-ADTYANATSDVL) and a shorter version of Peptide 5 of only six amino acid residues (TAMRA-YANATS). Fluorophore-labeled Peptide 5 was glycosylated 1.5 times faster than TAMRA-DANYTK and the short version was glycosylated 14 times faster (Table S2). Direct quantitative analysis using the TAMRA-YANATS peptide as the substrate (Fig. 2*E*) revealed a 3.5-fold lower apparent K_M_ and a 3.5-fold higher maximal initial turnover rate (Table 1): TAMRA-YANATS was over ten times more specific to the yeast OST3 complex than TAMRA-DANYTK. For further experiments, the TAMRA-YANATS peptide substrate was used.

**Table 1:** Kinetic parameters for peptide and LLO substrates with OST3 and OST6 complexes

Peptide parameters		K_M_ (μM)	k_cat_ (min^−1^)	k_cat_/K_M_ (min^-1^ μM^−s1^)
OST3 complex	TAMRA-DANYTK	201.0 ± 51.3	3.9 ± 0.6	0.02
	TAMRA-YANATS	55.6 ± 6.5	14.5 ± 0.7	0.26
OST6 complex	TAMRA-YANATS	44.8 ± 5.7	2.3 ± 0.2	0.05


Synthetic LLO (GlcNAc_2_-PP-Lipid) parameters					
	Lipid name	Lipid length			
OST3 complex	Citronellyl	C10	n.d.	n.d.	n.d.
	Farnesyl	C15	67.9 ± 6.7	1.7 ± 0.1	0.02
	Citronellylneryl	C20	20.6 ± 0.6	9.9 ± 0.06	0.48
	Citronellylfarnesyl	C25	12.5 ± 2.3	4.5 ± 0.3	0.36
OST6 complex	Citronellylneryl	C20	25.5 ± 1.7	1.9 ± 0.01	0.07

TAMRA, tetramethylrhodamine.

n.d. = activity not detected with TAMRA-DANYTK acceptor substrate.

**Figure 3:**
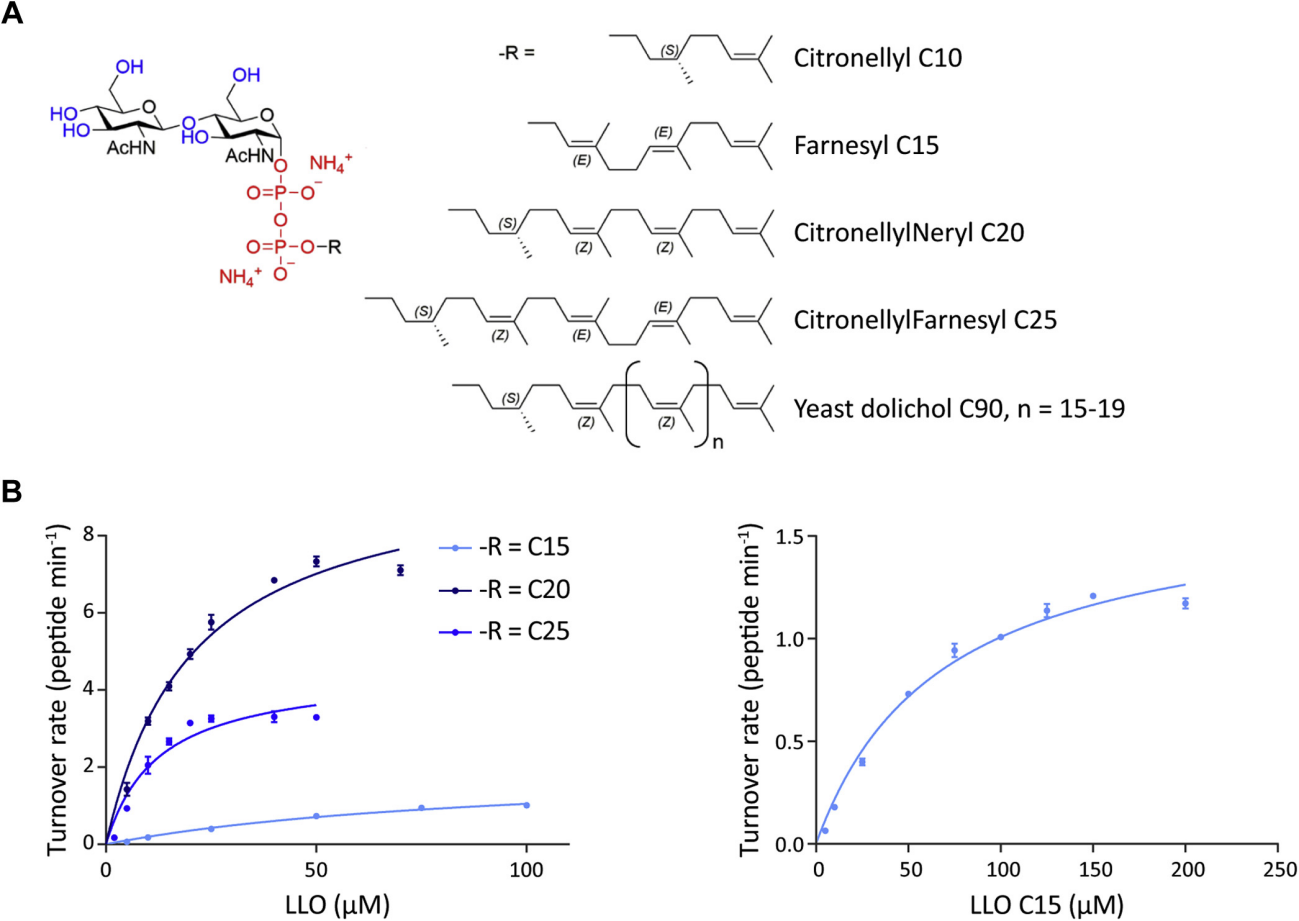
**Yeast OST can use synthetic LLO substrates to glycosylate peptides and displays different substrate preferences.***A*, structure of synthetic LLO substrates. Synthetic LLO substrates all have two GlcNAcs as the sugar moiety, followed by pyrophosphate and lipid moieties (−R) of variable stereochemistry and length, denoted by the carbon chain length. C10, (*S*)-citronellyl; C15, farnesyl; C20, (*S*)-citronellylneryl; C25, (*S*)-citronellylfarnesyl; C90, represents the natural yeast dolichol lipid tail structure where n = 15. Figure modified from Ramirez *et al.* 2017 (42). *B*, quantification of kinetic parameters for the synthetic LLOs. Reactions were performed with 0.1 μM purified OST3 complex, 25 μM peptide TAMRA-YANATS, and varying concentrations of the different synthetic LLOs. Glycopeptide product was measured by UPLC and turnover rates were determined from the linear initial phase of the reaction. The mean of three reactions is shown and the data were fitted by nonlinear regression according to the Michaelis–Menten formula (R^2^ = 0.9842 (LLO C15), 0.9685 (LLO C20), 0.9261 (LLO C25)) using Prism. Error bars indicate standard deviations from the mean (n = 3). The right panel shows the Michaelis–Menten fitting for LLO C15 across the full range of LLO C15 concentrations. The derived kinetic parameters are summarized in Table 1.

The apparent K_M_ and k_cat_ values were determined from data shown in Figures 2, *C* and *E*, 3*B*, and 5, *C* and *D*. Errors represent standard deviations from the mean of three replicates (n = 3) fitted by nonlinear regression using the Michaelis–Menten equation in Prism.

### Yeast OST has variable substrate specificity for synthetic LLO substrates

Yeast OST activity was measured using small chemically synthesized LLO analogs composed of a short polyprenyl chain linked *via* pyrophosphate to two GlcNAc residues, the smallest sugar entity transferred by the yeast OST (45, 46). The tested synthetic LLO analogs differed in their lipid moieties, varying in chain length and double-bond stereochemistry (Fig. 3*A*). Quantitative analysis of the LLO analog substrate specificity for the yeast OST3 complex revealed variable substrate specificity, similar to the single subunit kinetoplastid OST, TbStt3A (42) (Fig. 3*B*). The apparent K_M_ values shown in Table 1 revealed that the apparent affinity of the LLO substrate depended on the lipid carbon chain length, with the longest LLO, LLO C25, having the highest affinity and the shortest LLO, LLO C10, showing no detectable activity under the tested conditions. In line with this, the shortest LLO showing activity, LLO C15, was over 20 times less specific (k_cat_/K_M_ = 0.02 min^−1^ μM^−1^) than LLO C20 and LLO C25, with both a significantly lower turnover rate and affinity to the OST3 complex. It is important to note that LLO C15 also had an unsaturated C2 bond, which was saturated in LLO C20 and LLO C25. Although the apparent maximal turnover rates (k_cat_) increased with LLO lipid length, the highest turnover rates were observed with the second longest LLO, LLO C20, despite it exhibiting a lower affinity than LLO C25. LLO C20 was about 1/3-fold more specific than LLO C25 under our experimental conditions, suggesting a role of lipid conformation and isoprenoid chain length in substrate binding. Indeed, LLO C20 (*ZZ*) had a more similar double-bond stereochemistry to the natural yeast dolichol (*Z*_n_) than LLO C25 (*ZEE*), which may have accounted for its higher turnover rate, despite lower affinity when compared with the longer LLO C25.

### Synthetic nonhydrolyzable LLO analogs inhibit OST activity

**Figure 4:**
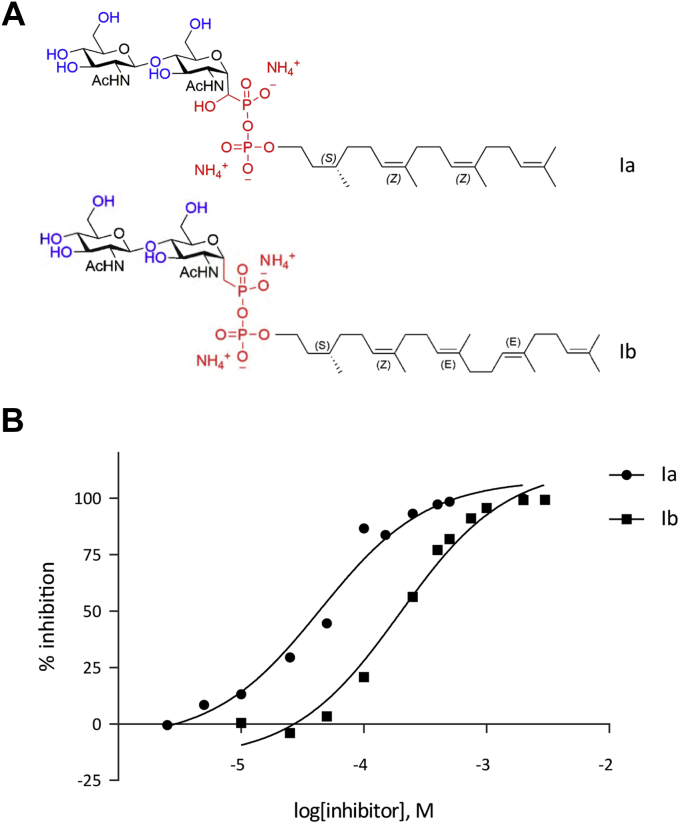
**Inhibition of yeast OST activity by synthetic nonhydrolyzable LLOs *in vitro*.***A*, structure of synthetic nonhydrolyzable LLOs. Synthetic nonreactive LLOs have two GlcNAcs as the sugar moiety, followed by pyrophosphonate and lipid moieties of variable stereochemistry and length, denoted by the carbon chain length. Ia, GlcNAc_2_-(OH)CPP-(*S*)-citronellylneryl (C20); Ib, GlcNAc_2_-CPP-(*S*)-citronellylfarnesyl (C25). Note that Ia is a hydroxy-phosphonate (with an additional OH group, single diastereomer, R/S configuration unassigned), while Ib is a phosphonate without an additional hydroxyl group. Figure modified from Ramirez *et al.* 2017 (42). *B*, quantification of inhibition by nonhydrolyzable LLOs shown in *A*. Reactions were performed with 0.1 μM purified OST3 complex, 25 μM peptide TAMRA-YANATS, 50 μM LLO C20, and varying concentrations of the different synthetic inhibitory LLOs. Glycopeptide product was measured by UPLC and turnover rates were determined from the linear initial phase of the reaction. % inhibition was determined relative to a control reaction without nonhydrolyzable LLOs. The data were fitted by nonlinear regression (R^2^ = 0.9778 (Ia), 0.9812 (Ib)) using Prism. The derived IC_50_ values are indicated in Table 2.

Nonhydrolyzable LLO substrate analogs containing an unreactive pyrophosphonate group have been shown to inhibit glycosylation of acceptor peptides by the single subunit TbStt3A (42). Since both TbStt3A and the yeast OST3 complex can use the same LLO analogs as substrates for glycosylation, we tested whether such nonreactive LLO analogs also inhibited glycosylation by the yeast OST3 complex. The nonreactive LLO analogs Ia and Ib had the same disaccharide and lipid structures as the reactive LLO substrates LLO C20 and LLO C25, respectively. However, they differed from their reactive counterparts in that, instead of a pyrophosphate, they contained either a nonhydrolyzable hydroxy-pyrophosphonate (Ia) or pyrophosphonate (Ib) moiety (Fig. 4*A*). Ib (called compound 34d in the TbStt3A study (42)) inhibited the yeast OST3 complex activity, but with a much lower apparent affinity (IC_50_ value of 198 μM) than that reported for TbStt3A (IC_50_ value of 26 ± 3 μM). With an IC_50_ value of 44 μM, Ia was a more potent inhibitor of the yeast OST3 complex than Ib (Fig. 4*B*, Table 2), in contrast to the apparent affinities of the reactive LLOs increasing with increased lipid length. Overall, both nonreactive LLO analogs exhibited lower apparent affinity (higher IC_50_) than the reactive hydrolyzable substrates (lower K_M_), which was in agreement with the observations of the study on TbStt3A (42).

**Table 2:** IC_50_ values of nonhydrolyzable synthetic LLO analogs

Nonhydrolyzable LLO	Lipid length	IC_50_ (μM)
GlcNAc_2_-(OH)CPP-(*S*)-citronellylneryl (Ia)	C20	44
GlcNAc_2_-CPP-(*S*)-citronellylfarnesyl (Ib)	C25	198

The apparent IC_50_ values of nonhydrolyzable LLO analogs were determined from data shown in Figure 4*B*.

### OST3 complex has a higher enzyme activity than the OST6 complex, despite similar affinities for both peptide and LLO substrates

**Figure 5:**
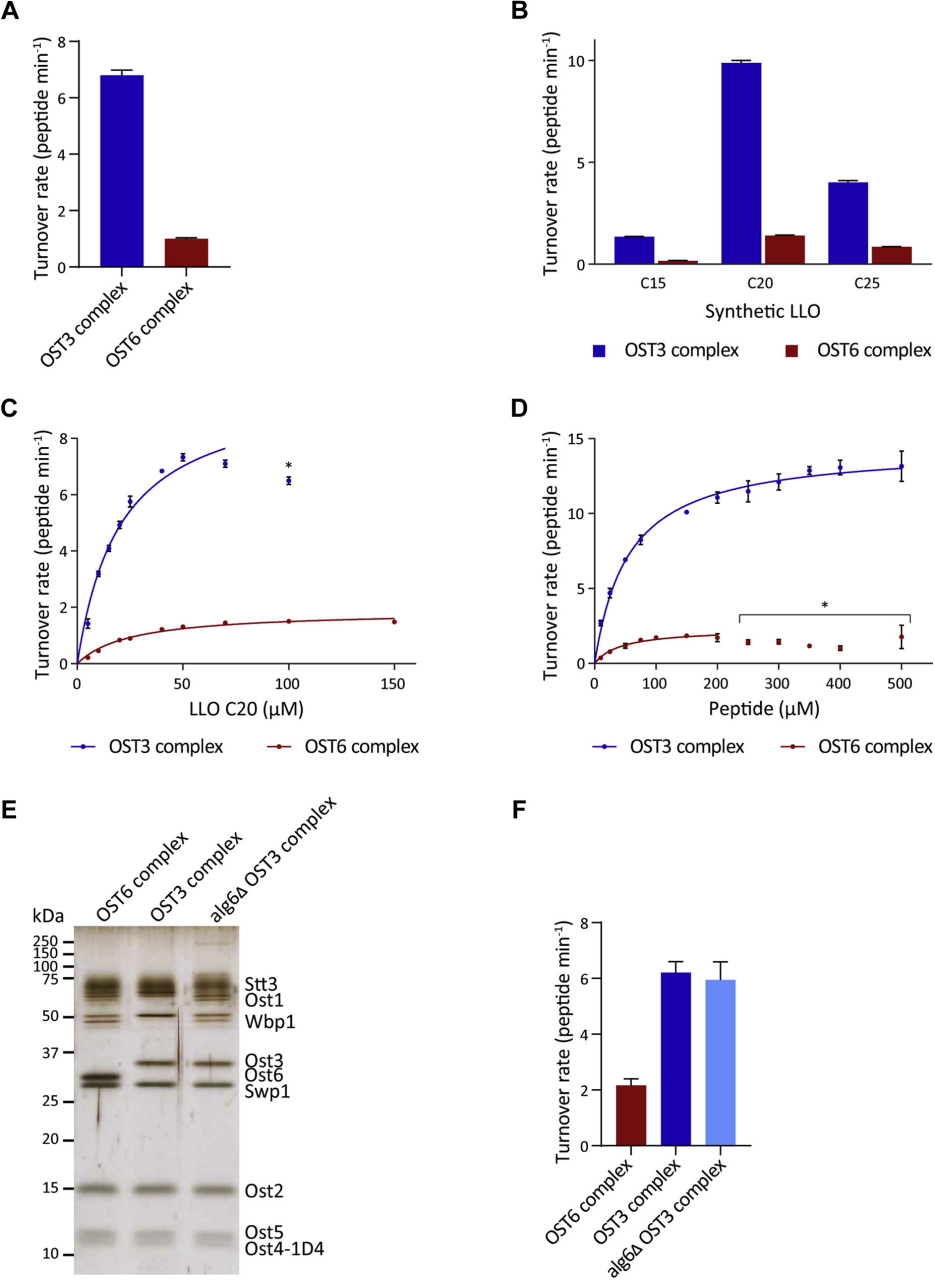
**OST3 complex turns over both glycosylation substrates faster than the OST6 complex, but with similar substrate affinities.***A*, enzyme activity of OST3 and OST6 complexes. Reactions were performed with 25 μM peptide TAMRA-YANATS, 50 μM LLO C20, and varying concentrations of purified OST3 complex (blue) or OST6 complex (*red*). Glycopeptide product was measured by UPLC and initial rates of reaction were determined by linear regression of the initial phase of the reaction. Turnover rates were determined by linear regression of the initial rates of reaction measured across the enzyme concentrations of 0.01–0.1 μM for OST3 complex and 0.1–0.5 μM for OST6 complex. Error bars indicate standard deviations from the mean of three reaction sets (n = 3). *B*, synthetic LLO preferences of OST3 and OST6 complexes. Reactions were performed with 25 μM peptide TAMRA-YANATS, 50 μM of the indicated LLO (C15, C20, or C25) and 0.09 μM of purified OST3 complex (*blue*) or 0.5 μM OST6 complex (*red*). Turnover rates were determined by linear regression of the initial phase of the reaction and accounting for the enzyme concentration in the reaction. Error bars indicate standard deviations from the mean of three reactions (n = 3). *C*, quantification of kinetic parameters for the synthetic LLO C20. Reactions were performed with 25 μM peptide TAMRA-YANATS, varying concentrations of synthetic LLO C20, and either 0.1 μM purified OST3 complex (*blue*) or 0.5 μM OST6 complex (*red*). Turnover rates were determined by linear regression of the initial phase of the reaction and accounting for the enzyme concentration in the reaction. The mean of three reactions is shown and the data were fitted by nonlinear regression according to the Michaelis–Menten formula (R^2^ = 0.9685 (OST3 complex), 0.9720 (OST6 complex)) using Prism. Error bars indicate standard deviations from the mean (n = 3). *Asterisk* indicates a data point not included in the Michaelis–Menten fitting, due to poor fitting. The derived kinetic parameters are summarized in Table 1. *D*, quantification of kinetic parameters for the peptide TAMRA-YANATS. Reactions were performed with 100 μM synthetic LLO C20, varying concentrations of peptide TAMRA-YANATS, and 0.1 μM purified OST3 complex (*blue*) or OST6 complex (*red*). Turnover rates were determined by linear regression of the initial phase of the reaction and accounting for the enzyme concentration in the reaction. The mean of three reactions is shown and the data were fitted by nonlinear regression according to the Michaelis–Menten formula (R^2^ = 0.9794 (OST3 complex), 0.9214 (OST6 complex)) using Prism. Error bars indicate standard deviations from the mean (n = 3). *Asterisk* indicates data points not included in the Michaelis–Menten fitting, due to poor fitting. The derived kinetic parameters are summarized in Table 1. *E*, SDS-PAGE (14 % acrylamide) silver staining of 0.2 μg purified OST3 complex, OST6 complex, and OST3 complex purified from *alg6Δ* cells. Multiple bands of Ost1p and Wbp1p represent the fully and hypoglycosylated forms of the proteins. Ost4p is tagged with the 1D4 epitope for purification. *F*, enzyme activity of OST complexes. Reactions were performed with 100 μM peptide TAMRA-YANATS, 50 μM LLO C20, and varying concentrations from 0.1 to 0.3 μM of purified OST3 complex, OST6 complex, or OST3 complex purified from *alg6Δ* cells. Turnover rates were determined as in *A* by linear regression of the initial rates of reaction across the enzyme concentrations of 0.1–0.3 μM for each OST. Error bars indicate the standard deviation from the mean turnover rate of three reaction sets (n = 3).

When comparing the enzyme activity of the OST3 complex with the OST6 complex *in vitro* using synthetic chitobiose-containing LLOs and the TAMRA-YANATS peptide, we found the OST6 complex activity to be drastically reduced as compared with the OST3 complex (Fig. 5*A*). The OST6 complex activity was also reduced compared with the OST3 complex using the TAMRA-DANYTK peptide (data not shown). To investigate whether the two OST complexes differed in substrate specificity, we tested all active LLO analogs in combination with the TAMRA-YANATS peptide and found a five- to eightfold reduction in peptide glycosylation activity for the OST6 complex compared with the OST3 complex (Fig. 5*B*). Both OSTs had the same trend in LLO analog specificity, with the highest turnover rates observed for LLO C20 and the lowest reaction rates for LLO C15. Quantitative analysis of the kinetic parameters for the OST6 complex with LLO C20 confirmed that the OST6 complex did indeed have a fivefold lower maximal turnover rate compared with the OST3 complex, despite very similar K_M_ values (Fig. 5*C*, Table 1). This illustrated that both OSTs have a similar binding affinity for this LLO substrate but differed in their efficiency of carrying out the glycosylation reaction. The same result was found when we analyzed the acceptor peptide TAMRA-YANATS: the OST6 complex peptide turnover rate was reduced six times as compared with that of the OST3 complex. However, the affinity for the peptide substrate was similar, as demonstrated by the K_M_ values (Fig. 5*D*, Table 1). Clearly, the two OST complex isoforms bound both the LLO and peptide substrates with similar affinity but differed in their glycosylation efficiency *in vitro*.

To determine whether hypoglycosylation of the OST subunits observed in the OST6 complex affected OST activity, we generated a hypoglycosylated OST3 complex by expressing it in a strain with a deletion in the LLO biosynthesis gene *ALG6.* This caused a global hypoglycosylation phenotype (47, 48). The purified OST3 complex from *alg6Δ* cells showed hypoglycosylation of OST subunits to a similar extent as the OST6 complex (Fig. 5*E*). Importantly, this hypoglycosylation of the OST3 complex did not affect the turnover rate of the enzyme (Fig. 5*F*). Therefore, the difference in glycosylation efficiency of the two OST isoforms appeared to depend on the presence of either the Ost3p or Ost6p subunit within the OST complex and not on the hypoglycosylation of subunits in the OST6 complex.

## Discussion

To obtain the two OST complex isoforms, we genetically tailored yeast strains. This modification of OST by expressing only one of the two alternative subunits allowed for the analysis of pure enzyme, but it also affected the glycosylation of proteins. Since some of the OST subunits are glycoproteins themselves, altering OST composition directly affected glycosylation of OST: we observed hypoglycosylation of Wbp1p in cells only expressing Ost6p, but site occupancy was not affected for the glycosylation sites of Ost1p and Stt3p, the two other glycoproteins of OST (Fig. 1*C*). To evaluate whether or not OST subunit hypoglycosylation affected OST activity as measured *in vitro*, we expressed the OST3 complex in an *alg6* mutant strain. Due to the incomplete assembly of the LLO substrate in this strain, a similar hypoglycosylation of Wbp1p as in an Ost6p-only strain was observed, but this hypoglycosylation did not affect OST activity *in vitro* (Fig. 5*F*). Therefore, we concluded that differential activities of the recombinant OST complexes were not due to differences in the concomitant changes of OST subunit glycosylation but rather reflected the effect of the OST3/6 subunit on OST function.

The detailed analysis of the N-glycan structures of OST subunits revealed additional information regarding the assembly and the cellular localization of the enzyme. We observed that the most abundant glycan on the three OST glycoproteins is Man_8_GlcNAc_2_ (40), most likely the result of N-glycan processing in the ER by glucosidases I and II and ER mannosidase I (49). Interestingly, both Wbp1p and Ost1p carried minor glycan structures with up to ten hexoses, indicative of either an incomplete processing by ER-localized hydrolases or an extension of the glycan by Golgi-localized mannosyltransferases such as Och1p (50, 51). We also observed N-glycan structures with a hexose number higher than 9 in OST generated in an *alg6* mutant strain (where only a Man_9_GlcNAc_2_ LLO is transferred) (data not shown), excluding the incomplete processing by ER hydrolases and confirming a transport of OST to early Golgi compartments. Indeed, the C-terminal, cytoplasm-localized KKXX signal of Wbp1p has been shown to mediate retrieval to the ER (52, 53). We therefore postulate that yeast OST can cycle between the ER and an Och1p-containing compartment.

The presence of a highly structured N-glycan on STT3 (N539 in yeast), close to the catalytic center, seems to be a property of multi-subunit eukaryotic OSTs. This glycosylation site is highly conserved (41), and an ordered N-glycan at this position has been found in yeast as well as in mammalian OST structures, thanks to the cryo-EM technology (13, 14, 31). We also note that this N-glycan is well protected in a groove of the OST complex that is formed by Stt3p, Wbp1p, and Swp1p. Irrespective of this shielding, this glycan was usually processed by ER-localized glucosidases and mannosidases, leading to the Man_8_GlcNAc_2_ oligosaccharide. We concluded that this processing had to occur before the assembly of OST.

Purification of OST allowed for a biochemical characterization of the enzyme. The peptide and LLO K_M_ values determined in this study were within range of those reported previously using tripeptide substrates and a dolichol-linked disaccharide LLO (54, 55). In our limited analysis of peptide substrate preference, we observed that the peptide sequence immediately surrounding the consensus glycosylation sequon N-X-(S/T) influenced peptide affinity and the rate of modification by OST. Based on the apo structure of yeast OST (13, 14) and peptide-bound structures of the human STT3B complex (31), as well as prokaryotic OSTs (6, 7, 10, 56), it was proposed that peptide substrate binding is highly conserved and involves direct interactions of the N and the S/T residues with conserved sites in the STT3/PglB/AglB proteins. Our experimental evidence suggests that a tyrosine residue is favorable at the -2 position of the acceptor peptide, which is in agreement with a previous report demonstrating that sequons with an aromatic residue at that position are glycosylated more frequently by the mammalian OST (57). In support of these findings, the structure of the peptide-binding site of the yeast OST reveals a cavity that can accommodate a larger amino acid side chain at the -2 position of the sequon (13).

Analysis of substrate specificity for the LLO lipid moiety demonstrated a dependence on the length of the isoprenoid lipid. Our set of LLO analogs tested in the *in vitro* reactions allowed us to postulate a preference for the longer isoprenoid lipids, although LLO C20 was preferred over the longer LLO C25, suggesting that lipid stereochemistry is also important for peptide turnover rates. LLO C15 was bound much less efficiently, but this substrate was not saturated at the first isopentenyl unit as in LLO C20 or LLO C25, a determining property of the eukaryotic dolichols (58).

Most importantly, the two yeast OST complex isoforms revealed a significant difference in overall substrate turnover rates *in vitro* while presenting similar affinities for the substrates used in our assays. The glycosylation rates by the OST6 complex were about five times slower than those of the OST3 complex when measured for both the peptide and LLO substrates, despite having similar substrate affinities for both the peptide and LLO. This finding corroborates with a previous report showing that an Ost3p-containing complex has a higher relative glycosylation activity than an Ost6p-containing complex *in vitro*, using an assay with LLO extract (39). Based on the available structures of eukaryotic OSTs (13, 14, 31), it is evident that the substrates used in our *in vitro* assay interact only with the common catalytic subunit, Stt3p, explaining the similar K_M_ values for the peptide and the LLO substrates for the two OST enzymes. Similarly, the catalytic activity is located exclusively in the STT3 subunit of OST, but the altered turnover rates measured for the two OST enzymes suggested an effect of the OST3/6 subunit on the catalytic cycle of the enzymes. Cryo-EM structures reveal that the OST3 subunit interacts directly with Stt3p *via* at least three of its four transmembrane helices (13, 14), an interaction also observed for the homologous MAGT1 in the mammalian STT3B complex (31). We therefore postulate that the interaction of the OST3/6 subunit with Stt3p affects the conformation or conformational changes of the catalytic subunit during the catalytic cycle of OST. We hypothesize that the OST3/6 C-terminal transmembrane domain is responsible for this modulating activity.

Conformational changes of the catalytic subunit during the catalytic cycle were shown to be critical for the activity of the bacterial homolog, PglB. Binding of the substrates leads to structural alterations, including ordering of the EL5 across both bound substrates and the active site (9, 10). Moreover, PglB makes several specific interactions with the LLO substrate, which implies that accurate alignment of the LLO in the binding pocket is required for an efficient glycosylation reaction (11). In light of our findings, Stt3p may adopt its active conformation at different rates, depending on whether Ost3p or Ost6p interacts with Stt3p.

Similar differences between turnover rates of different OST complexes were also reported for the human Stt3A and the Stt3B complexes and attributed to the different STT3 subunits in the two enzymes (25, 31). However, the two complexes also differ in the DC2/(MAGT1/TUSC3) subunits, paralogs of the yeast OST3/6 subunit. OST3/6 and DC2/(MAGT1/TUSC3) all directly interact with the catalytic STT3 subunits. It is possible that observed differences in Stt3A/Stt3B activity are not only due to the different STT3 subunits but also to a different regulation by the interacting DC2/(MAGT1/TUSC3) subunit.

Many organisms express two Stt3B-type OST complexes that differ in the incorporated oxidoreductase homolog. It was proposed that the oxidoreductase domains of these subunits have different protein substrate specificities, thereby modulating OST activity site specifically (34, 35, 36, 37). Here we show in yeast that the two OST complex isoforms with different oxidoreductases (Ost3p/Ost6p) also have different glycosylation turnover rates, independent of their protein substrate specificity. Therefore, we hypothesize that the Ost3p- or Ost6p-dependent differences in glycosite specificity observed *in vivo* are not solely due to the differences in the peptide-binding properties of the oxidoreductase domains of Ost3p and Ost6p, but, as shown with synthetic chitobiose-containing LLOs, are also a result of the intrinsically different rates of glycosylation by the two OST complex isoforms. Within the framework of this hypothesis, the protein sequence bound by the Ost3p/Ost6p subunit would define the timing of glycosylation of a nearby glycosylation site, indirectly modulating the folding rate of the glycoprotein. Further experiments that address the dual functionality of the yeast OST3/6 subunits are required to test this hypothesis, as well as whether it is a feature unique to yeast OSTs or also observed in oxidoreductase subunit paralogs of other organisms. As the gene duplication of OST3 homologs appears to have occurred independently in some fungi (including budding yeasts) and in vertebrates (33), the functionality may be different in different organisms.

## Experimental procedures

### Materials

All materials were purchased from Sigma unless specified otherwise.

### Yeast strain construction

Standard yeast media and genetic techniques were used for growth and strain construction (59, 60). Yeast strains used for OST expression and purification were derived from BY4742 and were described previously (13). Briefly, the OST3 complex strain (*MAT α his3Δ1 leu2Δ0 lys2Δ0 ura3Δ0 arg4Δ0 ost6::LEU2MX6 OST4-1D4::kanMX6* YEp352-*OST3*) and the OST6 complex strain (*MAT α his3Δ1 leu2Δ0 lys2Δ0 ura3Δ0 arg4Δ0 ost3::LEU2MX6 OST4-1D4::kanMX6* YEp352-*OST6*) were constructed so that either Ost3 or Ost6 is expressed. Using standard genetic techniques, the *OST3* or *OST6* genes were disrupted by replacement with the *Kluyveromyces lactis LEU2* gene (61) and the 1D4 epitope (62) was fused C-terminally to *OST4* by homologous recombination of a *1D4-kanMX6* cassette amplified by PCR from the pYM-1D4 plasmid using S2 and S3 primers (60, 63). pYM-1D4 was constructed by excising the 3Myc tag from pYM4 (60) by digestion using SalI and AsiSI restriction enzymes. The 1D4 tag sequence was inserted by ligating a similarly digested PCR product amplified from pYM4 using a forward primer containing the 1D4 tag sequence (5′-CGC GTC GAC GGT GGT TCC GGT GGT TCC TTG GAA GTT TTG TTT CAA GGT CCA ACT GAA ACT TCT CAA GTT GCT CCA GCT TAA GGC GCG CCA CTT CTA AAT AAG-3′) and the reverse primer 5′-GCG CCT GAG CGA GAC GAA ATA CG-3′. The C-terminal amino acid sequence fused to *OST4* is RTLQVDGGSGGS-LEVLFQGP-**TETSQVAPA** (linker region followed by a PreScission cleavage site underlined and the 1D4 epitope in bold). Correct tag integration was confirmed by PCR and DNA sequencing. Overexpression plasmids pOST3 (YEp352-*OST3* (23)) and pOST6 (YEp352-*OST6* (64)) were transformed and the strains were grown on standard synthetic dropout medium lacking uracil (SD-Ura) to retain the plasmid in the cells. To generate hypoglycosylated OST3 complex, the *ALG6* gene was deleted in the OST3 complex strain by homologous recombination of a NatNT2 cassette amplified by PCR from the pRS42N plasmid (65) using the forward primer 5′-AAT GGA CGG TGT CAG GAA TTC TTT TCT TCA CAT CAG GCT TCG CAT AGC AGC GAC ATG GAG GCC CAG AAT AC-3′ and the reverse primer 5′-TAT CAG TTG CGT CTG ACT GAC ATT GTA CAG TTA TAT AAG TTA AAA TGC GGT ATT TCA CAC CGC ACA GGT GTT GTC-3′. The resulting strain is *MAT α his3Δ1 leu2Δ0 lys2Δ0 ura3Δ0 arg4Δ0 ost6::LEU2MX6 OST4-1D4::kanMX6 alg6::NatNT2* YEp352-*OST3*.

### OST expression and purification

OST was expressed and purified based on the protocol described previously (13), with some modifications. 4× 1 L of yeast culture in 5 L flasks were grown at 30 °C, shaking at 180 rpm in SD-Ura medium (6.7 g/L yeast nitrogen base without amino acids (BD Difco), 20 g/L glucose, and 2 g/L SD amino acid supplement mix without uracil). Cells were harvested at OD_600_ 2–4 by centrifugation at 4000*g* for 10 min at 4 °C, and all steps hereafter were conducted at 4 °C. The cells were washed with cold ddH_2_O and resuspended in cold lysis buffer containing protease inhibitors (50 mM Hepes, pH 7.5, 150 mM NaCl, 1 mM MgCl_2_, 1 mM phenylmethanesulfonyl fluoride (PMSF), 20 tablets/L cOmplete EDTA-free protease inhibitor cocktail (Roche Diagnostics)). Glass beads (0.5 mm) were added and the cells were lysed in a bead beater (planetary mono mill, Pulverisette 6, Fritsch GmbH) at 400 power, 3× 4 min with 1 min pause between rounds. The lysate was separated from the beads using a 50 ml syringe, and then the unbroken cells were removed by centrifugation at 1500*g* for 15 min. Membranes were pelleted by centrifugation at 50,000*g* for 45 min, resuspended by douncing 1:1 (g:ml) in membrane storage buffer (50 mM Hepes, pH 7.5, 150 mM NaCl, 1 mM MgCl_2_, 35% (v/v) glycerol) and shock frozen in liquid nitrogen for storage at –80 °C.

Membranes were solubilized in solubilization buffer (50 mM Hepes, pH 7.5, 150 mM NaCl, 1 mM MgCl_2_, 1 mM MnCl_2_, 10% (v/v) glycerol, 1 mM PMSF, 20 tablets/L cOmplete EDTA-free protease inhibitor cocktail (Roche Diagnostics), 0.05 mg/ml DnaseI (from bovine pancreas), 1% (w/v) *n*-dodecyl-β-D-maltopyranoside (DDM) (Anatrace), and 0.2% (w/v) cholesteryl hemisuccinate (CHS)) in a ratio of 1:10 (membranes (g): buffer (ml)) for 1.5 h at 1.25 rpm on a rotation wheel. The insoluble fraction was removed by centrifugation at 50,000*g* for 45 min and the soluble supernatant was mixed with sepharose beads (CNBr activated sepharose 4B) coupled to Rho-1D4 antibody (University of British Columbia) equilibrated in purification buffer (50 mM Hepes, pH 7.5, 150 mM NaCl, 1 mM MgCl_2_, 1 mM MnCl_2_, 10% (v/v) glycerol, 0.03% (w/v) DDM, and 0.006% (w/v) CHS). After at least 3 h of incubation at 1.25 rpm on a rotation wheel, the 1D4 antibody-coupled beads were transferred onto a Protino filter column (Macherey-Nagel) and washed two times with ten column volumes of purification buffer. To elute the bound OST, the beads were incubated with purification buffer supplemented with 0.5 mg/ml 1D4 peptide (GenScript Corp.) for at least 2 h on the filter before being collected. The beads were washed with one column volume purification buffer. The eluted protein was concentrated at 3000*g* on an Amicon Ultra-15 Centrifugal Filter Device with a 100 kDa cutoff before purification by size-exclusion chromatography on a Superose 6 Increase 10/300 Gl column (GE Healthcare) to remove aggregates using purification buffer at a flow rate of 0.3 ml/min. The peak eluting at ∼14 ml was collected and concentrated again on an Amicon Ultra-15 ml Centrifugal Filter Device with a 100 kDa cutoff to a concentration of 2–4 mg/ml. The protein concentration was determined by BCA assay (Pierce, Thermo Fisher Scientific). The protein was either stored at 4 °C for a few days or shock frozen in liquid nitrogen and stored in aliquots at –80 °C. Gel samples were prepared by diluting the protein in 1× Lämmli supplemented with 1.5 M urea and incubated at 37 °C for 20 min. Gel samples were loaded on 14% acrylamide gels and SDS-PAGE and subsequent silver staining were performed using standard procedures.

### Sample preparation for site occupancy and glycan profile analysis by mass spectrometry

Purified OST samples collected after size-exclusion chromatography were prepared for mass spectrometry experiments using the filter-assisted sample preparation protocol (66): 50–100 μg of purified OST was denatured in UA buffer (8 M urea in 0.1 M Tris-HCl, pH 8.5) and loaded on an Amicon Ultra-0.5 ml Centrifugal Filter Device with a 30 kDa cutoff. The proteins were reduced with 50 mM dithiothreitol (DTT) in UA buffer for 1 h at 37 °C, washed with UA buffer, and subsequently alkylated with 65 mM iodoacetamine (IAA) in UA2 buffer (1 M urea in 0.1 M Tris-HCl, pH 8.5) for 1 h at 37 °C in the dark. The samples were washed three times with 0.05 M NH_4_HCO_3_ before protein digestion with 1.25 μg of sequencing-grade modified trypsin (Promega AG) per 100 μg protein sample in 0.05 M NH_4_HCO_3_ and incubated for 16 h at 37 °C. The peptides were eluted by centrifugation and dried. For site-occupancy analysis, the peptides were dissolved in 50 mM NaOAc, pH 5.2, and incubated with 1 μl of endoglycosidase H (EndoH, New England Biolabs) for 3 h at 37 °C. To ensure complete removal of the *N*-glycan, the reaction was boosted by addition of a subsequent 1 μl of EndoH and incubation for 16 h at 37 °C. The reaction was acidified to 0.5% formic acid (FA) and desalted using C18 ZipTips (Millipore) for mass spectrometric analysis. For glycoproteomic analysis, the peptides were directly dissolved in 2.5% acetonitrile (ACN)/0.1% FA and desalted using C18 ZipTips (Millipore).

### Site occupancy and glycan profile analysis by LC-MS/MS mass spectrometry

Samples were analyzed on a calibrated Q Exactive mass spectrometer (Thermo Fischer Scientific) coupled to a Waters nanoACQUITY UPLC System (Waters) with a Picoview nanospray source 500 model (New Objective). The tryptic samples were dissolved in 2.5% ACN/0.1% FA, loaded onto an Acclaim PepMap 100 trap column (75 μm × 20 mm, 100 Å, 3 μm particle size) and separated on a nanoACQUITY UPLC BEH130 C18 column (75 μm × 150 mm, 130 Å, 1.7 μm particle size), at a constant flow rate of 300 nl/min, with a column temperature of 50 °C and a linear gradient of 1 − 35% ACN/0.1% FA in 42 min, followed by a sharp increase to 98% acetonitrile in 2 min and then held isocratically for another 10 min. For site-occupancy analysis, one scan cycle comprised of a full-scan MS survey spectrum, followed by up to 12 sequential higher-energy collisional dissociation (HCD) scans based on the intensity. For site-occupancy analysis, full-scan MS spectra (400–2000 m/z) were acquired in the FT-Orbitrap at a resolution of 70,000 at 400 m/z, while HCD MS/MS spectra were recorded in the FT-Orbitrap at a resolution of 35,000 at 400 m/z. HCD MS/MS spectra were performed with a target value of 1e5 by the collision energy setup at a normalized collision energy (NCE) 25. For glycosylation profiling analysis, full-scan MS spectra (800–2000 m/z) were acquired in the FT-Orbitrap at a resolution of 70,000 at 400 m/z, while HCD MS/MS spectra were recorded in the FT-Orbitrap at a resolution of 35,000 at 400 m/z. HCD MS/MS spectra were performed with a target value of 5e5 by the collision energy setup at a NCE 22.

### Database analysis for site occupancy

MS and MS/MS data were processed into the Mascot generic format (mgfs) files and searched against the Swissprot database (version 202004) through Mascot engine (version 2.2) with the consideration of carbamidomethylation at cysteine, oxidation at methionine, and *N*-acetylhexosamine (HexNAc) at asparagine. The monoisotopic masses of 2+ or more charged peptides were searched with a peptide tolerance of 10 ppm and an MS/MS tolerance of 0.03 Da for fragment ions. Only peptides with a maximum of two missed cleavage sites were allowed in database searches. The false discovery rate (FDR) was 1%. The MS/MS validation was performed manually via Xcalibur 4.1. The list of masses of the peptides with and without HexNAc detected and analyzed is found in Table S3. For quantification, extracted ion chromatography (XIC) of peptides with and without HexNAc was plotted by their individual m/z with the mass tolerance of 10 ppm. Peak area was defined manually and integrated using Xcalibur 4.1. The site occupancy of each glycosylation site was calculated using the following equation:

$$\text{Site}-\text{occupancy}=\frac{\text{Peak}\;{\text{area}}_{\text{P}+\text{HexNAc}}}{\text{Peak}\;{\text{area}}_{\text{P}}+\text{Peak}\;{\text{area}}_{\text{P}+\text{HexNAc}}}\times 100$$

Peak area_P_ and Peak area_P + HexNAc_ are the areas under the curve measured for the unmodified peptide (P) and the peptide modified with HexNAc (P + HexNAc), respectively. The raw data, mgf, and results files were uploaded to the ProteomeXchange Consortium (67) *via* the PRIDE (68) partner repository with the dataset identifier PXD024590 and 10.6019/PXD024590.

### Database analysis for glycan profiling

For glycan profiling, the identification of each glycoform was first done by the Byonic 3.1 software (Protein Metrics). In brief, all raw data were loaded into Byonic and the search parameters were a peptide tolerance of 10 ppm and an MS/MS tolerance of 0.03 Da for fragment ions with the consideration of carbamidomethylation at cysteine, oxidation at methionine, and high mannose structures (Hex4-Hex12). Only peptides with a maximum of two missed cleavage sites and also semi-trypsin were allowed in database searches. The FDR was 1%. All assignments were confirmed manually with its corresponding MS/MS spectrum using Xcalibur 4.1. The list of masses of all glycoforms that were detected and analyzed is found in Table S4. For quantification, the data were processed as described in a previous study (69) using Xcalibur 4.1. In brief, the XIC of each glycoform was plotted by its individual m/z with the mass tolerance of 10 ppm. Peak area was defined manually and integrated using Xcalibur 4.1. The relative amount of each glycoform sharing the same peptide backbone was calculated using the following equation:

$$\text{Relative}\;\text{amount}\;\text{of each glycoform}\;\left(\%\right)=\frac{\text{Peak}\;\text{area}\;\text{of}\;\text{each}\;\text{glycoform}}{\text{Sum}\;\text{of}\;\text{peak}\;\text{areas}\;\text{of}\;\text{all}\;\text{glycoforms}}\times 100$$

### Chemical synthesis of LLO analogs and inhibitors

The chemical synthesis of all reactive LLO analogs and nonreactive LLO Ib was previously described in detail (42). Protected chitobiose diethyl hydroxy-phosphonate (minor diastereomer) and (*S*)-nerylcitronellyl phosphate were prepared according to the previously reported procedure (42). The synthesis of nonreactive LLO **Ia** (GlcNAc_2_-(OH)CPP-(*S*)-citronellylneryl (C20)) was also performed according to the reported procedure. In brief, benzyl and ethyl groups of the protected chitobiose diethyl hydroxy-phosphonate were removed by catalytic hydrogenation and treatment with excess TMSBr, respectively, to give deprotected chitobiose hydroxy-phosphonate (minor diastereomer, Fig. S3*A*). CDI-activation of (*S*)-nerylcitronellyl phosphate as phosphoroimidazolidate and a coupling reaction with deprotected chitobiose hydroxy-phosphonate allowed the isolation of the desired hydroxy-phosphonate nonhydrolyzable LLO analog **Ia** (minor diastereomer, Fig. S3*B*) after acetyl deprotection and purification by flash chromatography. Compound **Ia** (minor diastereomer) was obtained in pure form as a colorless lyophilisate (46.50 mg, 0.05 mmol, 36% over two steps), as confirmed by NMR spectroscopy and HRMS. Spectroscopic data to **Ia**: ^1^H NMR (400 MHz, MeOD) δ = 5.11–5.14 (m, 3H, H-6′, H-10′, H-14′), 4.49 (d, *J* = 8.4 Hz, 1H, H-5a), 4.34-4.37 (m, 2H, H-5b, H-7), 4.30 (dd, *J* = 9.2 Hz, 7.6 Hz, H-1a), 4.16-4.19 (m, 2H, H-2a, H-3a), 3.99-4.03 (m, 2H, H-1′), 3.90 (dd, *J* = 11.6 Hz, 1.6 Hz, H-6b1), 3.78-3.81 (m, 1H, H-6b2), 3.63-3.69 (m, 2H, H-6a1, H-4b), 3.58 (dd, *J* = 11.6 Hz, 6.8 Hz, 1H, H-6a2), 3.46-3.51 (dd, *J* = 10.8 Hz, 8.8 Hz, 1H, H-3b), 3.39 (t, *J* = 8.0 Hz, 1H, H-4a), 3.32-3.35 (m, 2H, H-1b, H-2b), 1.99-2.09 (m, 16H, H-5′, H-8′, H-9′, H-12′, H-13′, 2xNHAc), 1.96-1.98 (m, 1H, H-4′a), 1.68 (s, 9H, H-18′, H-19′, H-20′), 1.62 (s, 3H, H-17′), 1.34-1.47 (m, 3H, H-2′, H-4′b), 1.11-1.21 (m, 1H, H-3′), 0.93 (d, *J* = 6.4 Hz, 3H, H-16′). ^13^C NMR (101 MHz, MeOD) δ = 173.9, 173.7 (2xs, 2xNHC=OCH_3_), 136.2 (s, C-7′), 135.8 (s, C-11′), 132.3 (s, C-15′), 126.7 (s, C-14′), 126.2 (s, C-10′), 125.4 (s, C-6′), 103.1 (s, C-5a), 82.6 (s, C-4a), 78.1 (s, C-1b), 76.7 (s, C-5b), 75.8 (s, C-3b), 73.3 (d, *J* = 1.3 Hz, C-7), 72.1 (s, C-2b), 71.9 (s, C-1a), 71.9 (s, C-3a), 65.6 (d, *J* = 5.9 Hz, C-1′), 62.6 (s, C-6a), 62.6 (s, C-6b), 57.6 (s, C-4b), 54.2 (d, *J* = 9.2 Hz, C-2a), 38.9 (s, C-2′), 38.8 (s, C-3′), 38.8 (s, C-4′), 33.2 (s, C-8′), 30.8 (s, C-12′), 30.6 (s, C-17′), 27.7 (s, C-5′), 26.4 (s, C-9′), 26.0 (s, C-13′), 23.8 (s, C-19′), 23.7 (s, C-20′), 23.2 (s, NHC=OCH_3_), 22.8 (s, NHC=OCH_3_), 19.7 (s, C-16′), 17.8 (s, C-18′). ^31^P NMR (122 MHz, MeOD) δ = 9.7 (d, *J* = 25.4 Hz, P1), -8.8 (d, *J* = 25.4 Hz, P2). ESI-HRMS (-) *m/z* calculated 871.3764 (M-[H^+^]), found 871.3786 for C_37_H_65_N_2_O_17_P_2_^−^.

Concentrations of reactive LLO analogs were determined by *in vitro* glycosylation (see below) by titrating varying amounts of LLO against a known amount of fluorescently labeled peptide. Concentrations of nonreactive LLO analogs were determined by *in vitro* glycosylation by titrating varying amounts of nonreactive LLO while using known amounts of both fluorescently labeled peptide and active LLO analog.

### *In vitro* glycosylation assay

TAMRA-labeled peptides with 5-TAMRA at the N-terminus and an amide group at the C-terminus were synthesized by WatsonBio Sciences. Unlabeled peptides (Table S1) were synthesized by Genscript Corp. and were designed so that the asparagine of the glycosylation sequon was in the middle (except for Peptide 8). Lyophilized peptides were dissolved in dimethyl sulfoxide (DMSO), unless specified otherwise, to a 3 mM stock solution and further diluted in ddH_2_O for reactions.

Reactions were performed in a total volume of 10 μl containing the specified amount of TAMRA-labeled peptide, synthetic LLO analog, 10 mM MnCl_2_, and purified OST after size-exclusion chromatography in purification buffer (50 mM Hepes, pH 7.5, 150 mM NaCl, 1 mM MgCl_2_, 1 mM MnCl_2_, 10% (v/v) glycerol, 0.03% (w/v) DDM, and 0.006% (w/v) CHS). The concentration of OST was calculated using the molecular weight of 280 kDa. The reaction mixture was preincubated at 30 °C for 5 min before adding the peptide to start the reaction that proceeded at 30 °C. Time points were taken such that the reaction was in the linear phase. At each time point, 1 μl reaction mixture was taken and the reaction was stopped by dilution in stop solution (0.1% FA in 10% ACN, 10 mM KPO_4_ pH 8.0) such that the final peptide concentration was 2 μM.

Samples were analyzed by reverse-phase chromatography using a UPLC Dionex UltiMate 3000 with an Accucore 150-C18 100 × 2.1 mm 2.6 μm column (Thermo Fisher Scientific). One microliter sample (2 pmol of peptide) was injected and measured by isocratic elution using a certain proportion of ACN (15% ACN for peptide TAMRA-DANYTK; 20% ACN for peptide TAMRA-YANATS) in 10 mM KPO_4_, pH 8.0 buffer so that the glycopeptide and peptide are fully separated and elute within 3 min at a constant flow rate of 0.7 ml/min, with a column temperature of 45 °C. The peptide and glycopeptide were detected through TAMRA fluorescence (excitation wavelength: 546 nm; emission wavelength: 579 nm).

The UPLC profiles were analyzed by integrating the peaks corresponding to the glycopeptide and peptide using Chromeleon 7 (Dionex) and calculating the amount of glycopeptide formed using the following equation, as previously described (43):

$$\left[\text{GP}\right]=\frac{\text{Peak}\;{\text{area}}_{\text{GP}}}{\text{Peak}\;{\text{area}}_{\text{P}}+\text{Peak}\;{\text{area}}_{\text{GP}}}\times \left[\text{P}\right]$$

[GP] is the concentration of glycopeptide, Peak area_GP/P_ is the area under the curves measured for the glycopeptide or peptide, respectively, and [P] is the concentration of peptide at the start of the reaction. Time points were taken such that the reaction was in the linear phase and the data were fitted using GraphPad Prism 8 by linear regression to determine initial rates of reaction. Unless specified otherwise, turnover rates were determined from the initial rates of reaction by taking into account the enzyme concentration in the reaction. For determination of K_M_ and k_cat_ values, the data were fitted by nonlinear regression using the Michaelis–Menten equation using GraphPad Prism 8.

For quantification of the kinetic parameters for peptide TAMRA-DANYTK, reactions were performed with 2.8 μg purified extract containing OST3 complex before size exclusion (corresponds to approximately 1 μM OST3 complex), 150 μM LLO C20, and varying concentrations (10–400 μM) of the peptide. For TAMRA-YANATS, reactions were performed with 0.1 μM purified OST3 complex, 100 μM LLO C20, and varying concentrations (10–500 μM) of the peptide. For quantification of the kinetic parameters for the different LLO analogs, reactions were performed with 0.1 μM purified OST3 complex, 25 μM peptide TAMRA-YANATS, and varying concentrations of the different synthetic LLO analogs (5–200 μM for LLO C15; 5–150 μM for LLO C20; 2–50 μM for LLO C25). Turnover rates and kinetic parameters were determined as described above.

For the peptide competition assay to screen for a better peptide substrate, reactions were performed with 0.4 μM purified OST3 complex, 150 μM LLO C20, 25 μM TAMRA-DANYTK, and 25 μM unlabeled peptide (Genscript Corp.). The amount of glycosylated TAMRA-DANYTK peptide formed after 30 min was measured and inhibition was determined as a percentage relative to a control reaction without unlabeled peptide added.

For quantification of inhibition by synthetic nonreactive LLO analogs, reactions were performed with 0.1 μM purified OST3 complex, 25 μM peptide TAMRA-YANATS, 50 μM LLO C20, and varying concentrations of the different synthetic nonreactive LLOs (2.5–2000 μM for Ia; 2.5–3000 μM for Ib). Initial reaction rates were determined as described above and inhibition was determined as a percentage relative to the reaction rate of a control reaction without nonreactive LLO added. Data were fitted by nonlinear regression using GraphPad Prism 8 to determine the IC_50_ values.

For comparison of OST3 complex and OST6 complex activity, reactions were performed with 25 μM peptide TAMRA-YANATS and 50 μM LLO C20. In total, 0.1–2 μM of purified OST3 complex or OST6 complex was added to the reaction by adding the amount of protein in microgram that corresponds to the desired enzyme concentration, using the same molecular weight of 280 kDa for both complexes. Turnover rates were determined as the slope from the linear regression of the initial rates of reaction measured across the enzyme concentrations of 0.01– 0.1 μM for OST3 complex and 0.1–0.5 μM for OST6 complex. For comparison of OST3 complex, OST6 complex, and OST3 complex purified from *alg6Δ* cells, reactions were performed with 100 μM peptide TAMRA-YANATS, 50 μM LLO C20, and varying concentrations of purified OST complex (0.1–0.3 μM). Turnover rates were determined as the slope from the linear regression of the initial rates of reaction measured across the enzyme concentrations of 0.1–0.3 μM for each OST. For quantification of the kinetic parameters for the OST6 complex, reactions were performed with the same conditions as for the OST3 complex, except that 0.5 μM purified OST6 complex was used in reactions for determining the kinetic parameters for LLO C20. For comparison of synthetic LLO preferences, reactions were performed with 25 μM peptide TAMRA-YANATS, 50 μM of the indicated LLO (LLO C15, C20 or C25), and either 0.09 μM of purified OST3 complex or 0.5 μM OST6 complex. Turnover rates were determined as described above and the enzyme concentration was taken into account.

## Data availability

All data are contained within the article and the supporting information. The mass spectrometry proteomics data have been deposited to the ProteomeXchange Consortium *via* the PRIDE partner repository with the dataset identifier PXD024590 and 10.6019/PXD024590.

## Supporting information

This article contains supporting information.

## Conflict of interest

The authors declare that they have no conflicts of interest with the contents of this article.
