# Supplementary material for: Substrate specificities and reaction kinetics of the yeast oligosaccharyltransferase isoforms
Source: J Biol Chem. 2021 May 21;296:100809. doi: 10.1016/j.jbc.2021.100809 (PMC8191290; doi:10.1016/j.jbc.2021.100809)
Supplement: Tables S1–S4 and Figures S1–S3 [file mmc1.pdf]

Supporting Information

Substrate specificities and reaction kinetics of the yeast oligosaccharyltransferase isoforms

Jillianne Eyring[1], Chia-Wei Lin[1,2], Elsy Mankah Ngwa[1], Jérémy Boilevin[3], Giorgio Pesciullesi[3], Kaspar P. Locher[4], Tamis Darbre[3], Jean-Louis Reymond[3], Markus Aebi[1*]

[1]Institute of Microbiology, Department of Biology, ETH Zürich, 8093 Zürich, Switzerland
[2]Functional Genomics Center Zürich, University of Zürich/ETH Zürich, 8057 Zürich, Switzerland
[3]Department of Chemistry and Biochemistry, University of Berne, 3012 Bern, Switzerland
[4]Institute of Molecular Biology and Biophysics, Department of Biology, ETH Zürich, 8093 Zürich, Switzerland

*Corresponding author: Markus Aebi
E-Mail: markus.aebi@micro.biol.ethz.ch

Supp. Table 1. Library of unlabeled peptides derived from yeast sequences

Peptide #	Peptide name	Peptide a.a. sequence	Solvent used
Peptide 1	Pdi site 1 12mer	TLVEKNITLAQI	DMSO
Peptide 2	Pdi site 2 12mer	KNSDVNNSIDYE	DMSO
Peptide 3	Pdi site 3 12mer	PAYLANETFVTP	DMSO
Peptide 4	Pdi site 4 12mer	IDADFNATFYSM	DMSO
Peptide 5	Pdi site 5 12mer	ADTYANATSDVL	DMSO
Peptide 6	Pdi site 1 6mer	EKNITL	DMSO
Peptide 8	Pdi site 1 20mer	EYVKAAETLVEKNITLAQID	3 % ammonia*
Peptide 9	Cpy 1	LGIDPNVTQYTG	DMSO
Peptide 10	Cpy 2	SQKVRNWTASIT	DMSO
Peptide 12	Wpb1	LSPSGNDSETQY	DMSO
Peptide 13	Gpi12	RVRELNESAALL	DMSO
Peptide 14	Sec66	TKFSNNGTFFET	DMSO
Peptide 15	Gpi13	ILEPLAKNISNT	DMSO
Peptide 16	Gas1	FFYSNNGSQFYI	DMSO
Peptide 17	Stt3	DNNTWNNTHIAI	DMSO
Peptide 18	Rot1	TYSRYNQTETFK	DMSO
Peptide 19	Gpi16	GAPLFNSTEKLY	DMSO
Peptide 20	Plb2	SDAGFNISLSDL	DMSO

DMSO, dimethyl sulfoxide; *separate control reaction used containing 3 % ammonia

Supp. Table 2. Turnover of synthetic TAMRA-labeled peptides

Peptide	Glycopeptide detected	Turnover after 5 min (%)	Relative turnover (%)
TAMRA-DANYTK	Yes	6	100
TAMRA-YANATS	Yes	84	1400
TAMRA-ADTYANATSDVL	Yes	10	167

TAMRA, tetramethylrhodamine

Turnover of TAMRA-labeled peptides after 5 min was determined using 0.5 µM OST3 complex, 20 µM indicated peptide and 150 µM LLO C20. Relative turnover compared to TAMRA-DANYTK is given by defining TAMRA-DANYTK turnover as 100 %.

Supp. Table 3. Peptide and glycopeptide masses used for site-occupancy analysis by mass spectrometry

Protein	Glycosite	Peptide sequence N* = glycosylated asparagine	Peptide [M+H]+	z	m/z	Peptide + HexNAc [M+H]+	z	m/z
Ost1	N99	VSFFSAYFTNEATFLNSQLLAN*STTAPGDDGESEIR	3899.844	4	975.711	4102.922	4	1026.480
	N217	FSSN*ETLAIVYSHNAPLNQVVNLR	2686.417	3	896.139	2889.485	3	963.828
	N400	DSPVPFTN*VSIETQK	1546.819	2	773.909	1749.898	2	875.449
	N400	FTN*VSIETQK	1020.621	2	510.811	1222.621	2	611.811
Stt3	N539	TTLVDNNTWN*NTHIAIVGK	2111.106	3	704.369	2314.185	3	772.062
Wbp1	N60	LEYLDIN*STSTTVDLYDKEQR	2503.237	3	835.079	2706.315	3	902.772
	N332	LTLSPSGN*DSETQYYTTGEFILPDR	2804.340	3	935.447	3007.419	3	1003.140

N*, glycosylated asparagine; $[M+H]^+$, mass of singly protonated molecule; z, ion charge; m/z, mass to charge ratio; HexNAc, *N*-acetylhexosamine.

Supp. Table 4. Glycopeptide masses used for glycan profile analysis by mass spectrometry

Protein	Glycosite	Peptide sequence N* = glycosylated asparagine	Peptide [M+H]+	Glycan composition + $HexNAc_2$	Glycan [M+H]+	Glyco-peptide [M+H]+	z	m/z
Ost1	N99	VSFFSAYFTNEATFLNSQLLAN*STTAPGDDGESEIR	3899.825	Hex7	1540.540	5440.365	4	1360.847
			3899.825	Hex8	1702.576	5602.418	4	1401.360
			3899.825	Hex9	1864.646	5764.471	4	1441.874
			3899.825	Hex10	2026.704	5926.529	4	1482.388
			3899.825	Hex11	2188.760	6088.585	4	1522.902
			3899.825	Hex12	2350.816	6250.641	4	1563.416
	N217	FSSN*ETLAIVYSHNAPLNQVVNLR	2686.395	Hex7	1540.540	4226.935	3	1409.650
			2686.395	Hex8	1702.593	4388.988	3	1463.668
			2686.395	Hex9	1864.646	4551.041	3	1517.686
			2686.395	Hex10	2026.695	4713.090	3	1571.702
			2686.395	Hex11	2188.760	4875.155	3	1625.724
			2686.395	Hex12	2350.816	5037.211	3	1679.742
Stt3	N539	TTLVDNNTWN*NTHIAIVGK	2111.088	Hex7	1540.540	3651.628	3	1217.881
			2111.088	Hex8	1702.593	3813.681	3	1271.899
			2111.088	Hex9	1864.646	3975.734	3	1325.917
			2111.088	Hex10	2026.703	4137.791	3	1379.936
			2111.088	Hex11	2188.788	4299.876	3	1433.964
			2111.088	Hex12	2350.848	4461.936	3	1487.984
Wbp1	N60	NYKLEYLDIN*STSTTVDLYDK	2495.219	Hex7	1540.540	4035.759	3	1345.925
			2495.219	Hex8	1702.593	4197.812	3	1399.943
			2495.219	Hex9	1864.646	4359.865	3	1453.960
			2495.219	Hex10	2026.702	4521.921	3	1507.979
			2495.219	Hex11	2188.788	4684.007	3	1562.008
			2495.219	Hex12	2350.848	4846.067	3	1616.028
	N332	LTLSPSGN*DSETQYYTTGEFILPDR	2804.326	Hex7	1540.540	4344.867	3	1448.961
			2804.326	Hex8	1702.593	4506.919	3	1502.978
			2804.326	Hex9	1864.646	4668.972	3	1556.996
			2804.326	Hex10	2026.702	4831.029	3	1611.015
			2804.326	Hex11	2188.788	4993.114	3	1665.043
			2804.326	Hex12	2350.848	5155.175	3	1719.063

N*, glycosylated asparagine; $[M+H]^+$, mass of singly protonated molecule; z, ion charge; m/z, mass to charge ratio; HexNAc, *N*-acetylhexosamine; Hex#, number of hexoses (either glucose or mannose).

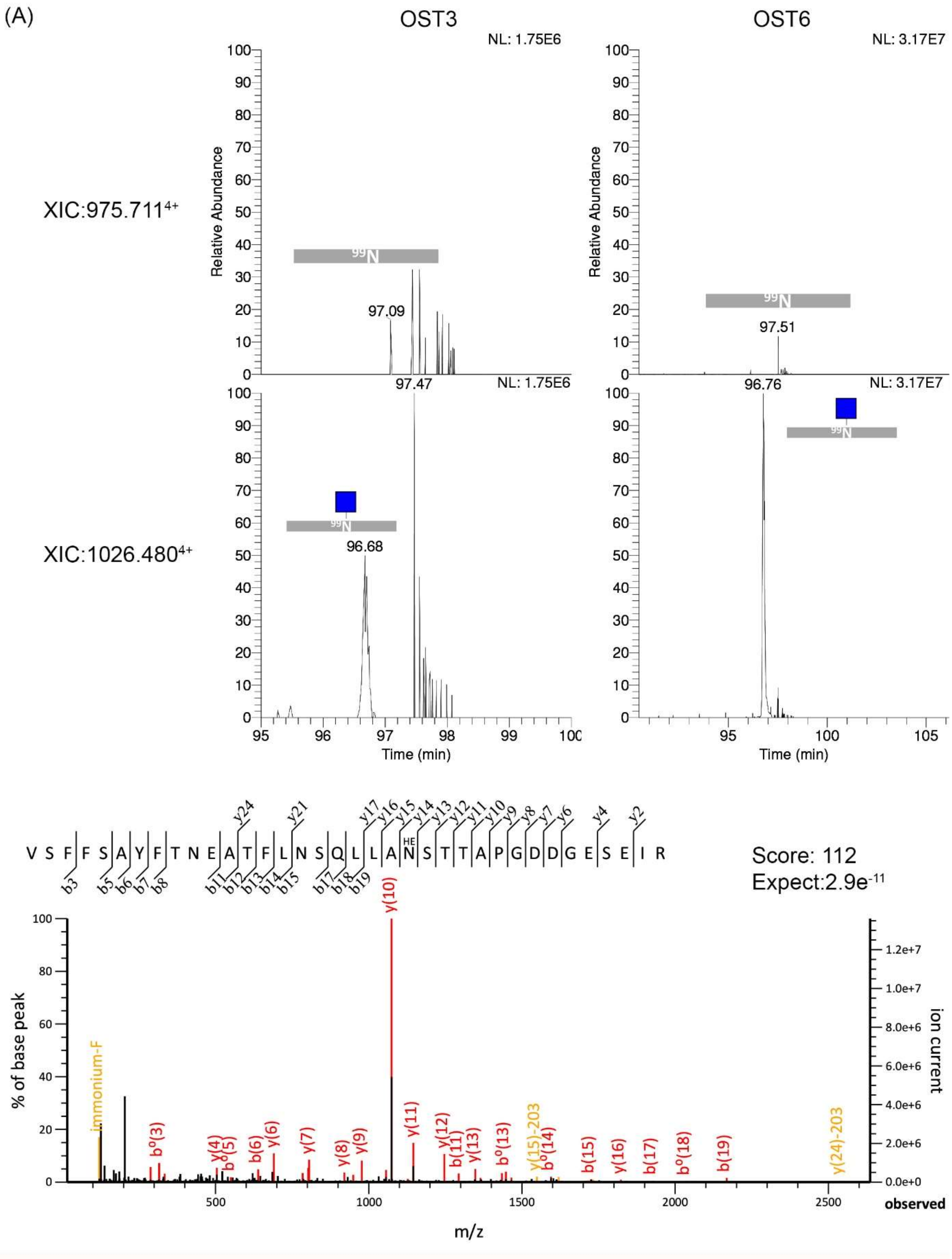

(A)
OST3
OST6
XIC:975.711⁴⁺
XIC:1026.480⁴⁺
NL: 1.75E6
NL: 3.17E7
Relative Abundance
97.09
97.51
97.47
96.76
96.68
Time (min)
V S F F S A Y F T N E A T F L N S Q L L A N S T T A P G D D G E S E I R
Score: 112
Expect:2.9e-11
% of base peak
ion current
m/z
observed
immonium-F
y(10)
y(15)-203
y(24)-203

(B)

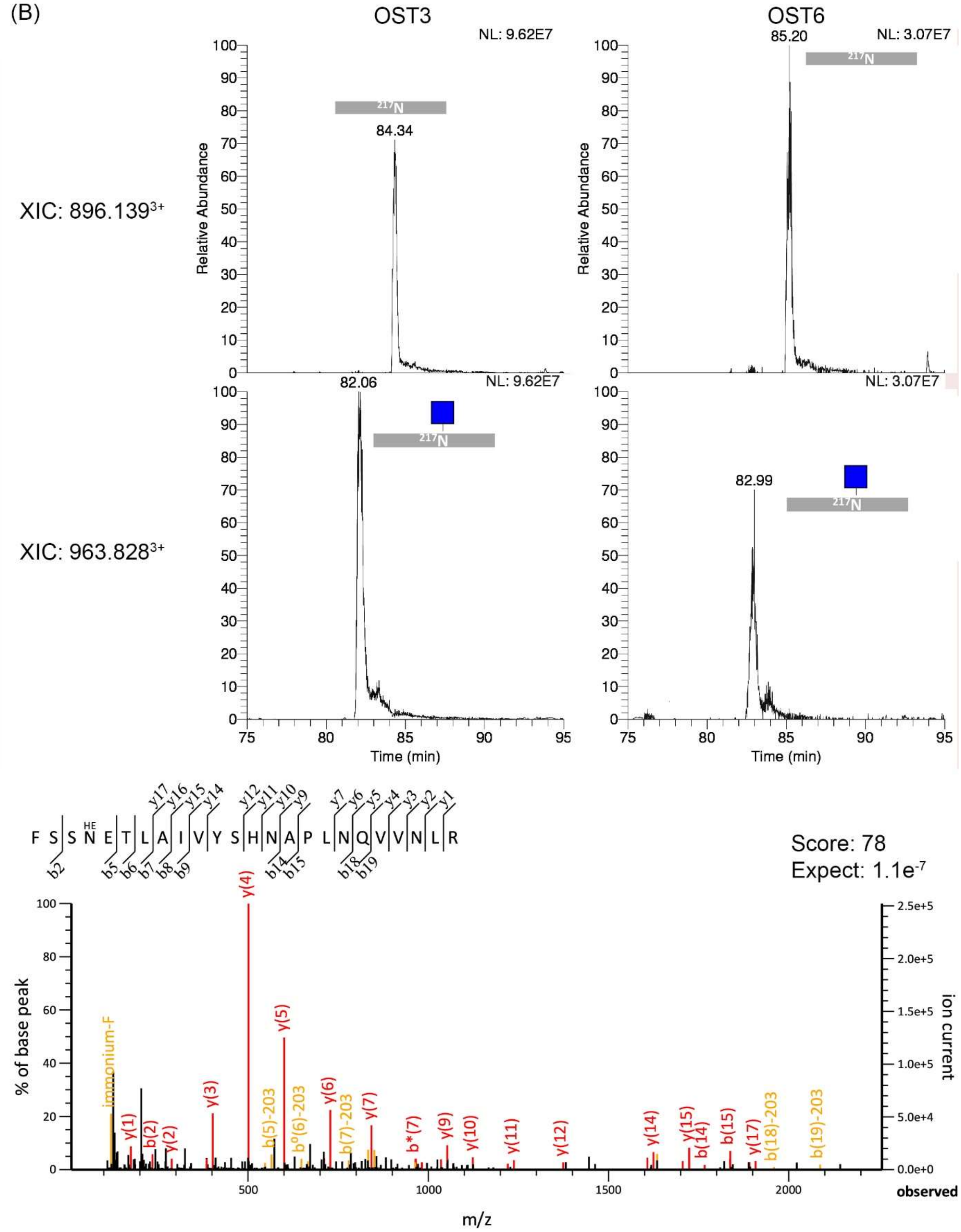
OST3
OST6
NL: 9.62E7
NL: 3.07E7
217N
84.34
85.20
82.06
82.99
XIC: 896.139 3+
XIC: 963.828 3+
Relative Abundance
Time (min)
F S S N E T L A I V Y S H N A P L N Q V V N L R
HE
Score: 78
Expect: 1.1e-7
% of base peak
ion current
m/z
observed
immonium-F
y(1)
b(2)
y(2)
y(3)
y(4)
b(5)-203
y(5)
b⁰(6)-203
y(6)
b(7)-203
y(7)
b*(7)
y(9)
y(10)
y(11)
y(12)
y(14)
y(15)
b(14)
b(15)
y(17)
b(18)-203
b(19)-203

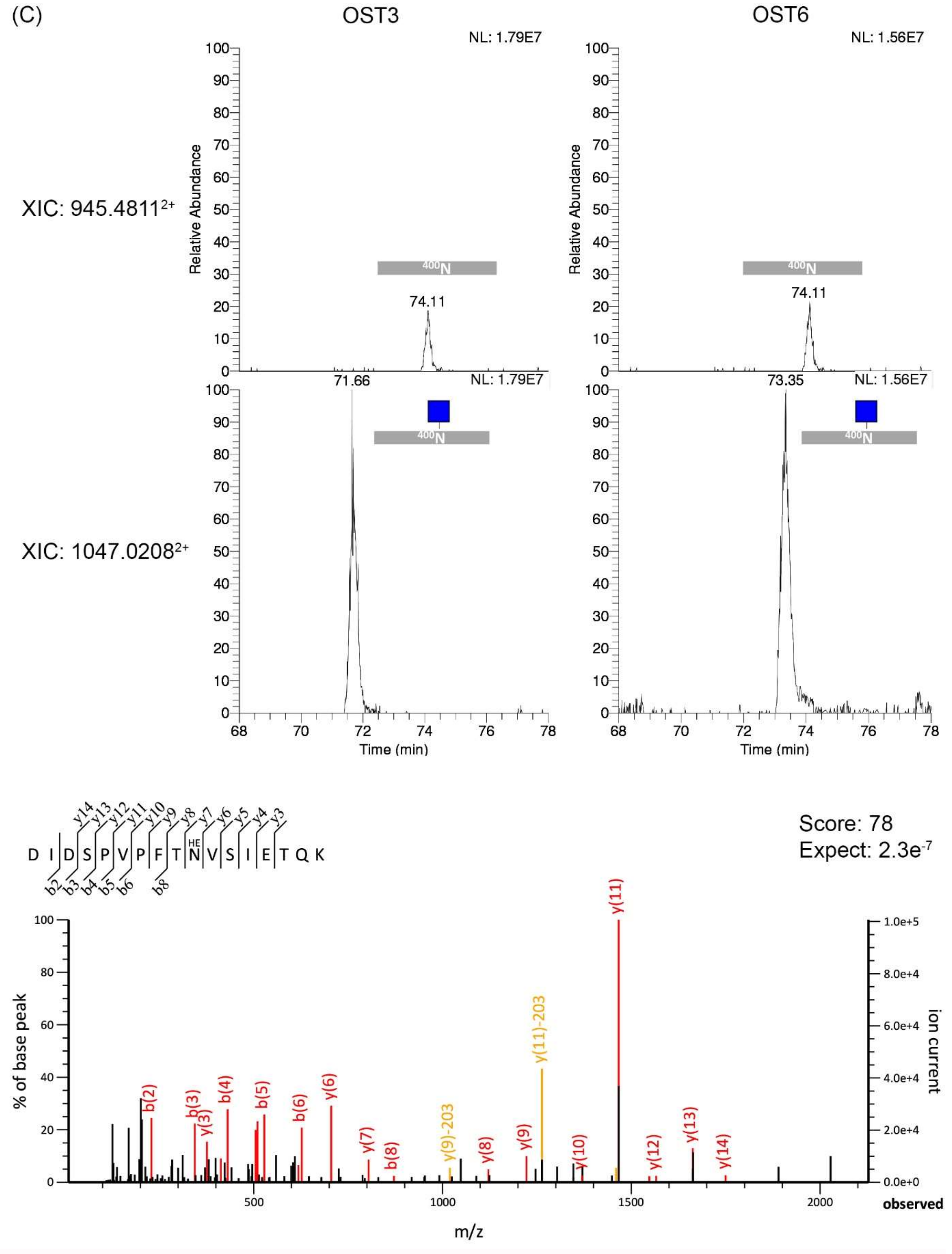
(C)
OST3
OST6
XIC: 945.4811[2+]
XIC: 1047.0208[2+]
NL: 1.79E7
NL: 1.56E7
Relative Abundance
74.11
71.66
73.35
Time (min)
Score: 78
Expect: 2.3e-7
D I D S P V P F T N V S I E T Q K
% of base peak
ion current
m/z
observed

(D)

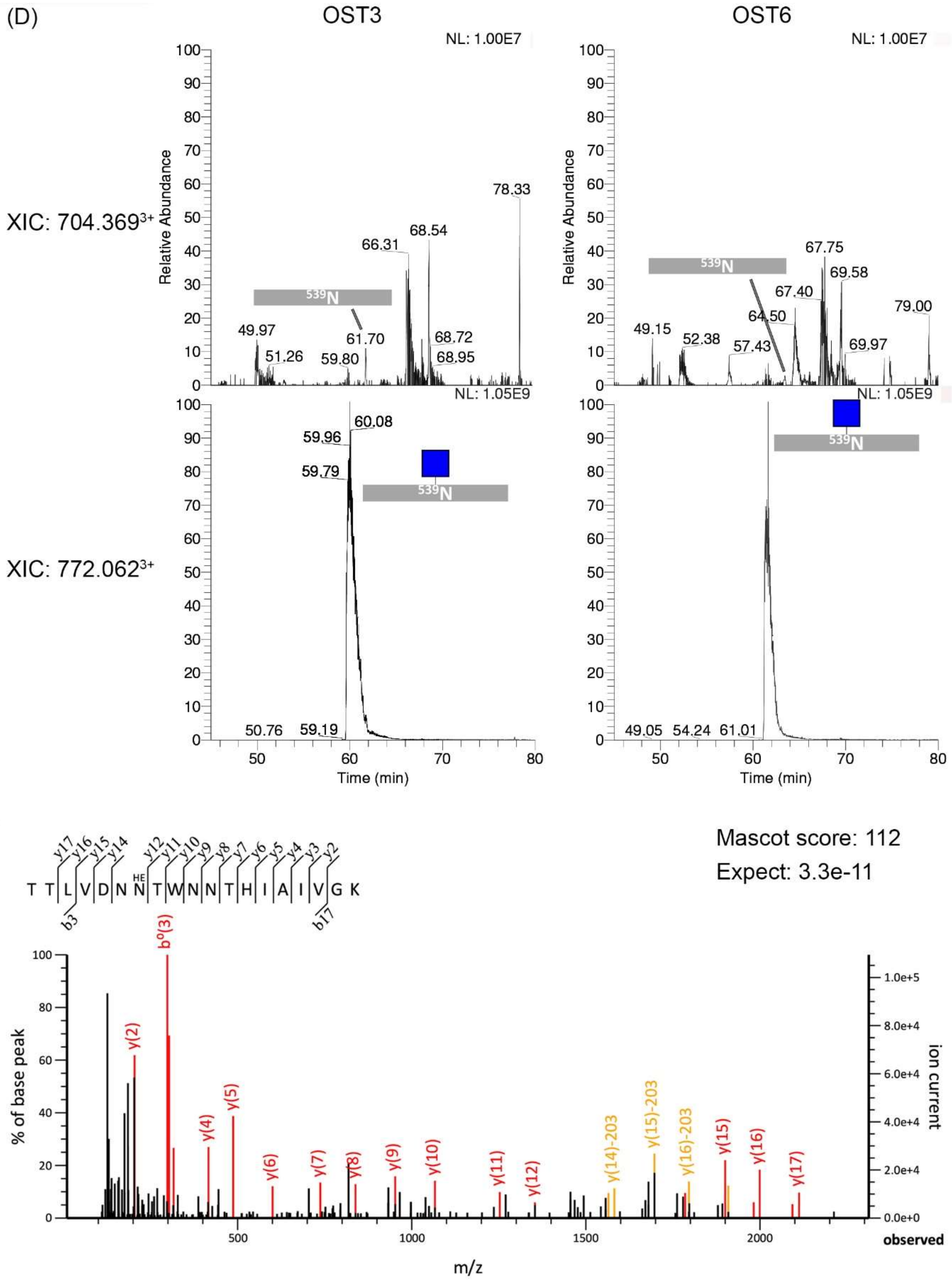

OST3
OST6
NL: 1.00E7
NL: 1.05E9
XIC: 704.369³⁺
XIC: 772.062³⁺
Relative Abundance
Time (min)
539N
78.33
68.54
66.31
49.97
51.26
59.80
61.70
68.72
68.95
67.75
69.58
67.40
64.50
79.00
49.15
52.38
57.43
69.97
60.08
59.96
59.79
50.76
59.19
49.05
54.24
61.01
Mascot score: 112
Expect: 3.3e-11
T T L V D N N T W N N T H I A I V G K
% of base peak
ion current
m/z
observed
b°(3)
y(2)
y(4)
y(5)
y(6)
y(7)
y(8)
y(9)
y(10)
y(11)
y(12)
y(14)-203
y(15)-203
y(16)-203
y(15)
y(16)
y(17)

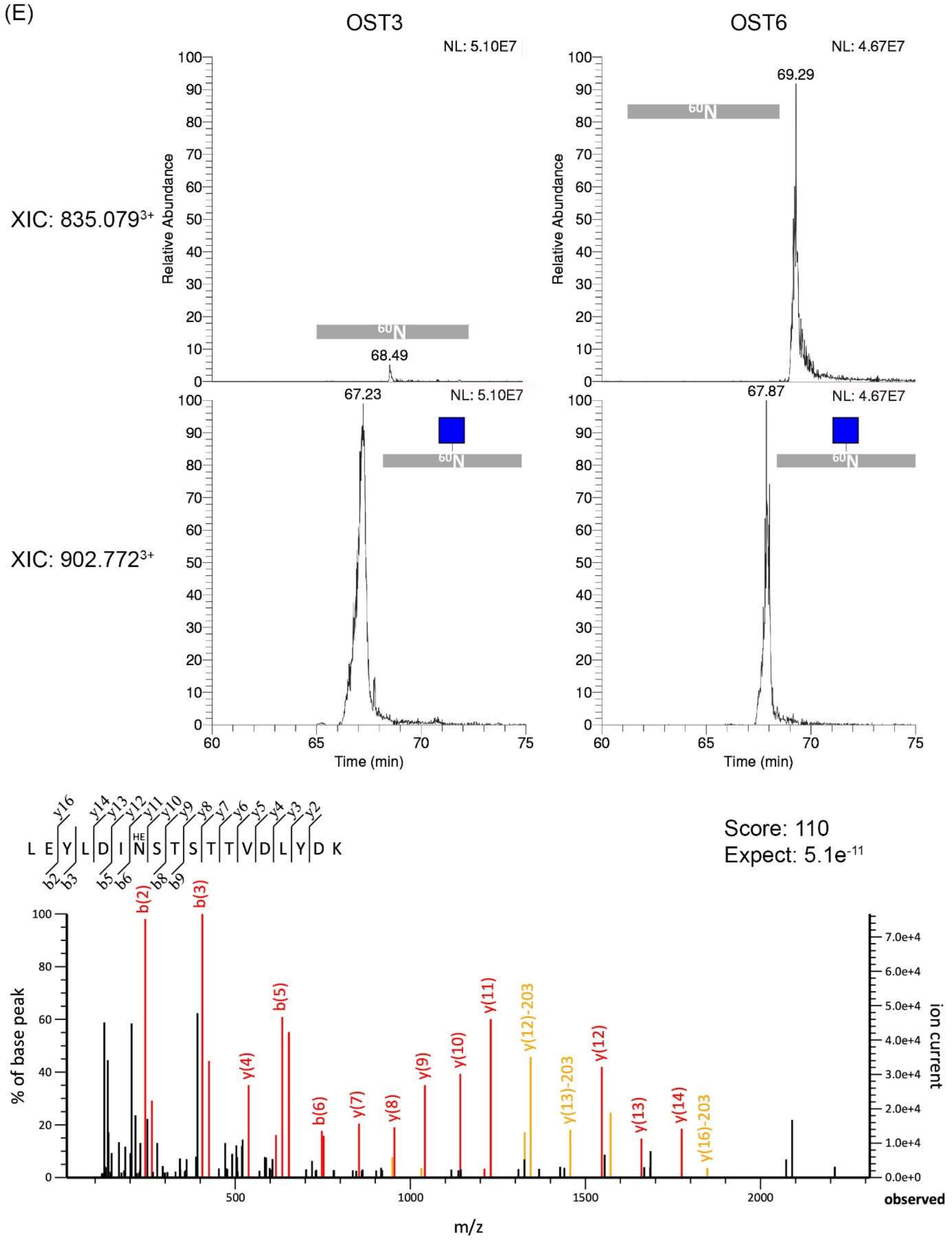
(E)
OST3
OST6
XIC: 835.079³⁺
XIC: 902.772³⁺
NL: 5.10E7
NL: 4.67E7
68.49
69.29
67.23
67.87
Relative Abundance
Time (min)
Score: 110
Expect: 5.1e-11
L E Y L D I N S T S T T V D L Y D K
% of base peak
ion current
m/z
observed

(F)

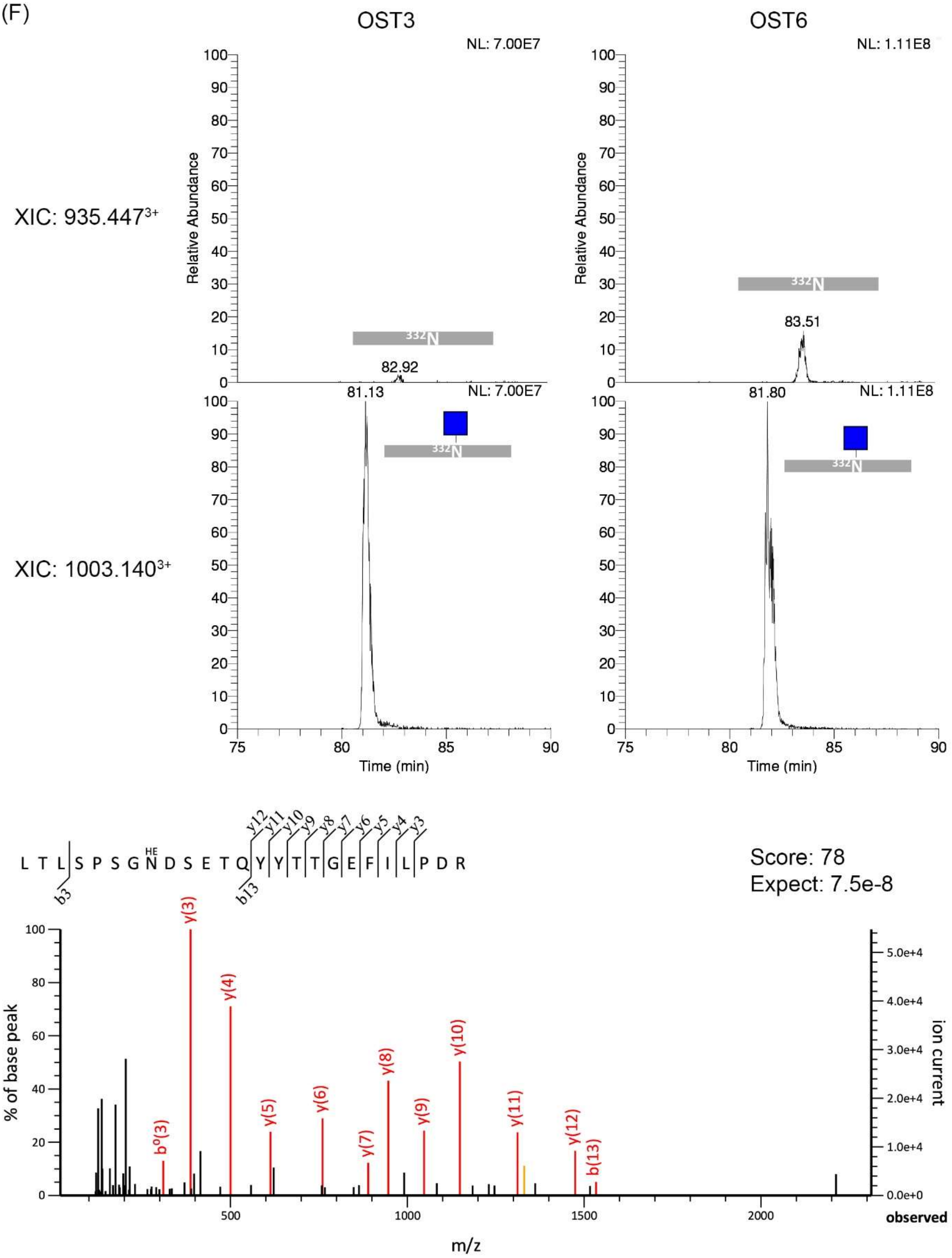

Supp. Figure 1 (to Figure 1C). Site-occupancy analysis of the OST subunits.
The extracted ion chromatography (XIC) of the tryptic peptide with and without HexNAc (blue square) from **(A)** N99 of Ost1p, **(B)** N217 of Ost1p, **(C)** N400 of Ost1p, **(D)** N539 of Stt3p, **(E)** N60 of Wbp1p and **(F)** N332 of Wbp1p are shown for the OST3 complex (OST3) and the OST6 complex (OST6). The m/z of the peptides with and without HexNAc are summarized in Supp. Table 3. The mass tolerance of XIC was 10 ppm. The corresponding MS/MS spectra from Mascot results are shown in the lower panel of each figure together with the score and the expected P value. HE represents HexNAc on the indicated asparagine (N).

(A)

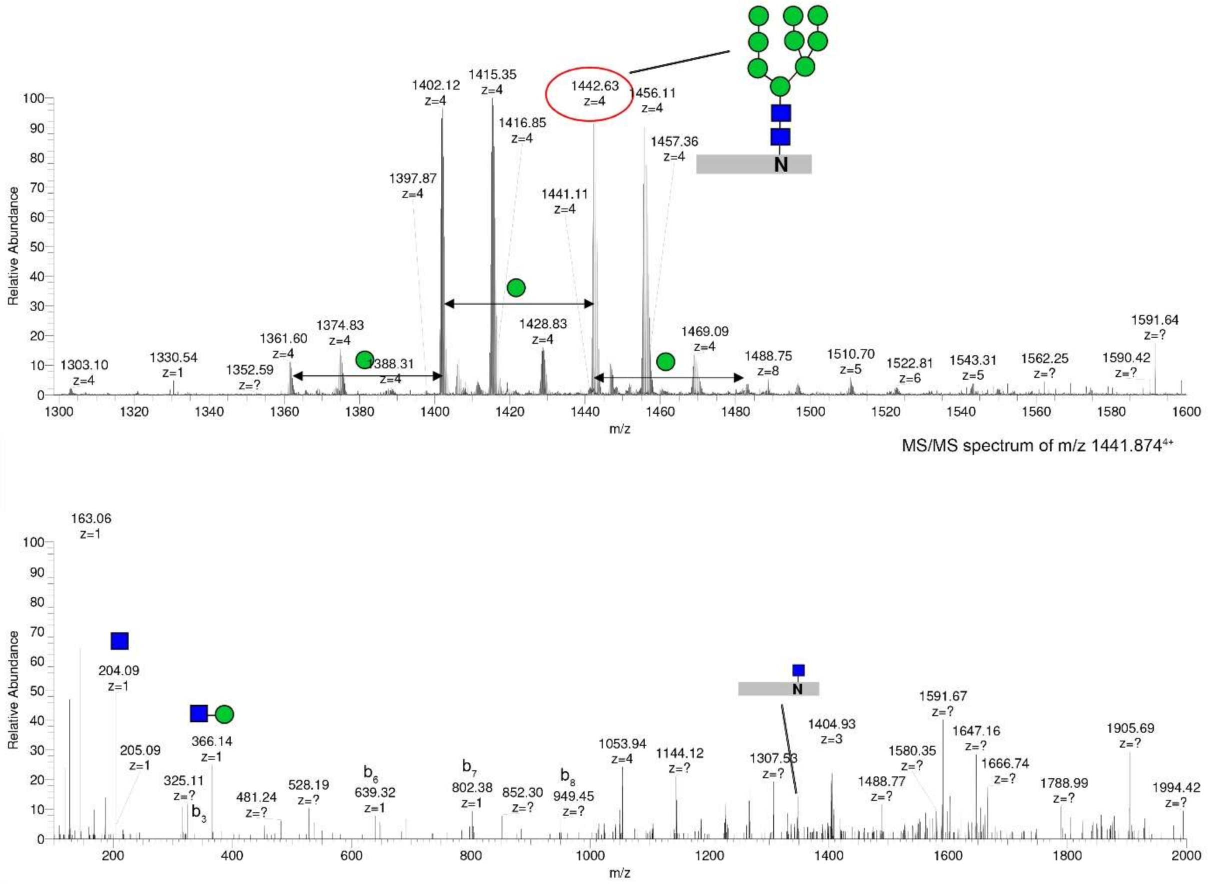
Relative Abundance
m/z
MS/MS spectrum of m/z 1441.874^{4+}

(B)

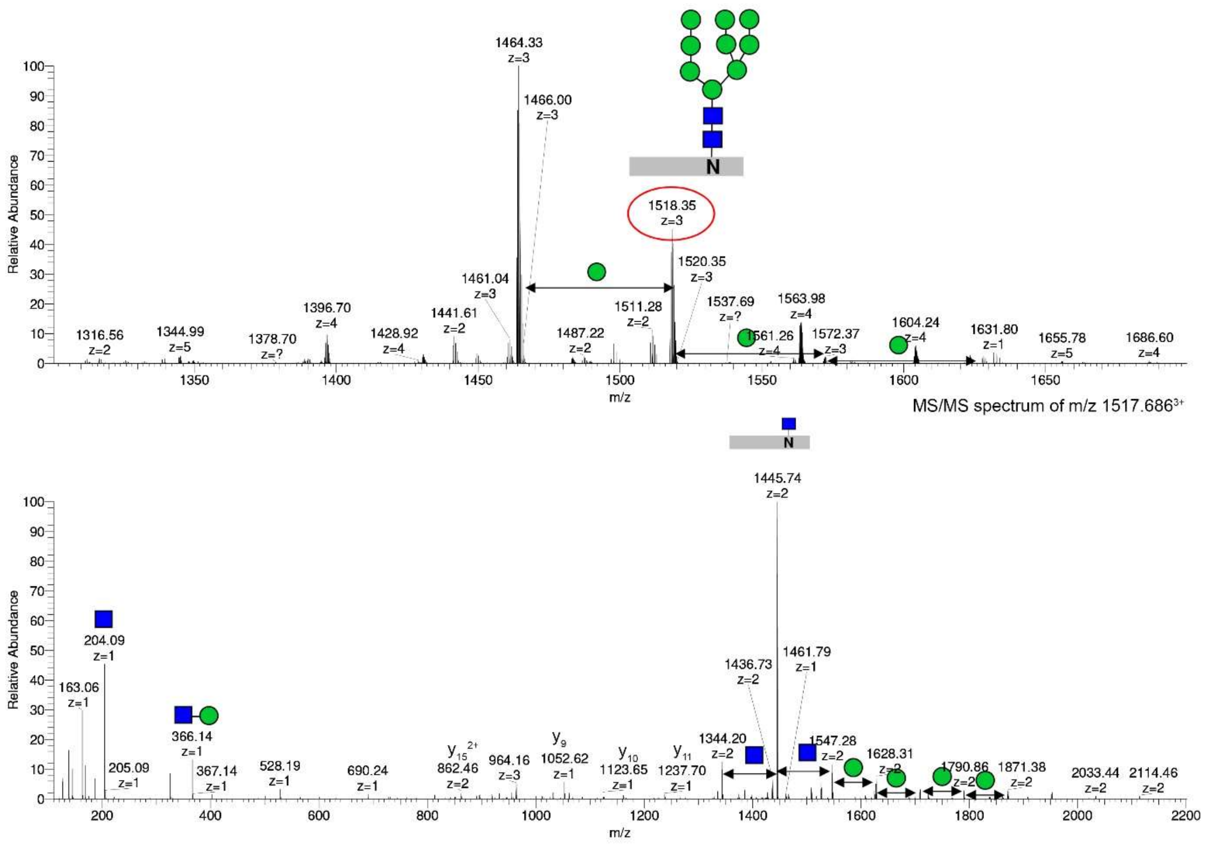
Relative Abundance
m/z
MS/MS spectrum of m/z 1517.686^{3+}

(C)

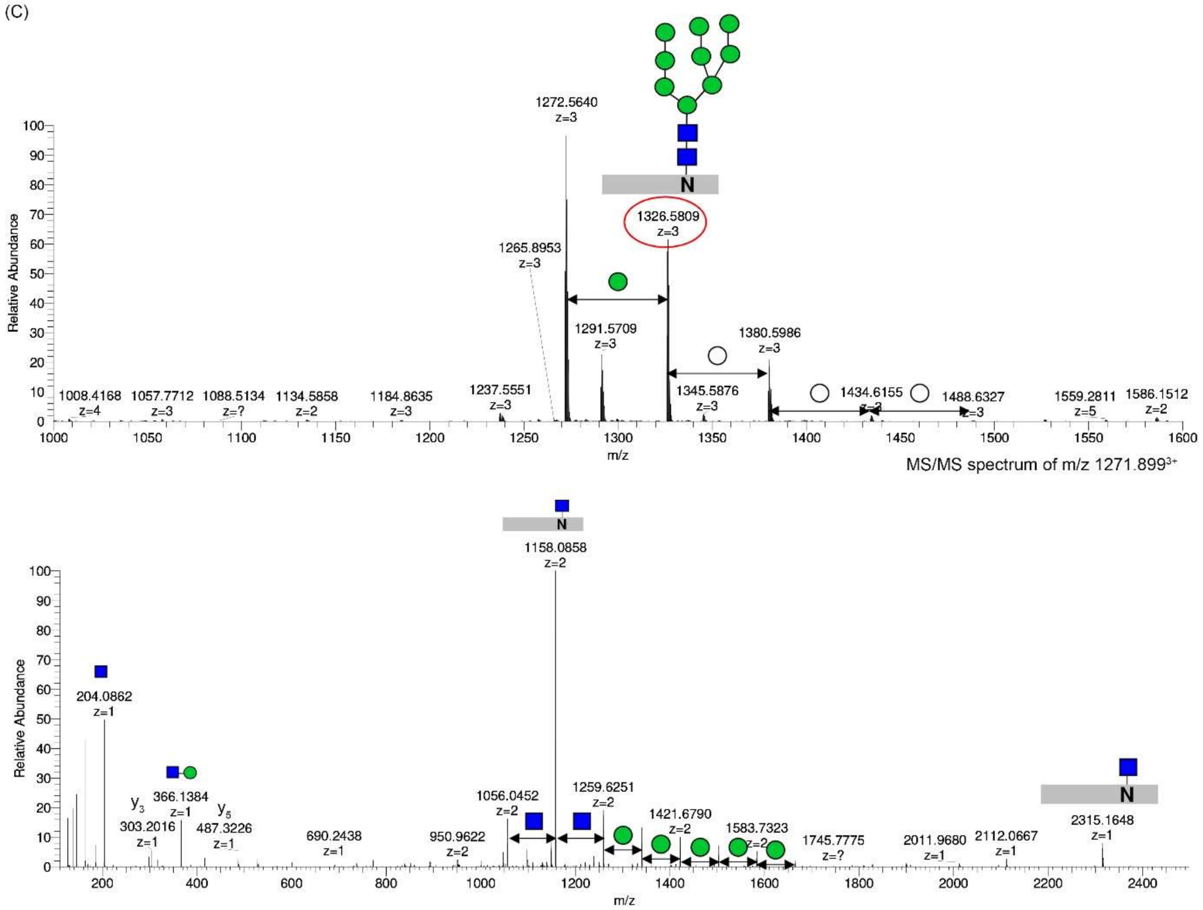
1272.5640
z=3
N
1326.5809
z=3
1265.8953
z=3
1291.5709
z=3
1380.5986
z=3
1008.4168
z=4
1057.7712
z=3
1088.5134
z=?
1134.5858
z=2
1184.8635
z=3
1237.5551
z=3
1345.5876
z=3
1434.6155
1488.6327
z=3
1559.2811
z=5
1586.1512
z=2
Relative Abundance
m/z
MS/MS spectrum of m/z 1271.899³⁺
1158.0858
z=2
204.0862
z=1
366.1384
z=1
y3
303.2016
z=1
y5
487.3226
z=1
690.2438
z=1
950.9622
z=2
1056.0452
z=2
1259.6251
z=2
1421.6790
z=2
1583.7323
z=2
1745.7775
z=?
2011.9680
z=1
2112.0667
z=1
2315.1648
z=1

(D)

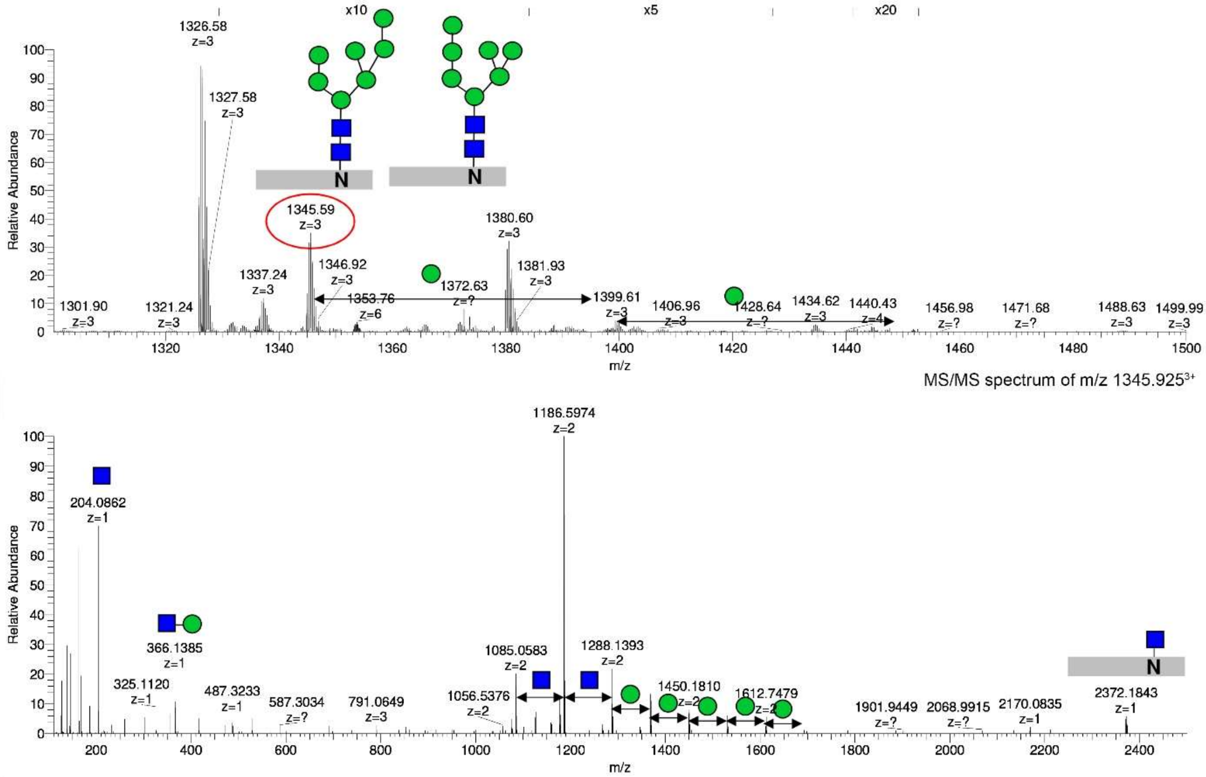
x10
x5
x20
1326.58
z=3
1327.58
z=3
N
1345.59
z=3
1380.60
z=3
1337.24
z=3
1346.92
z=3
1372.63
z=?
1381.93
z=3
1353.76
z=6
1399.61
z=3
1301.90
z=3
1321.24
z=3
1406.96
z=3
1428.64
z=?
1434.62
z=3
1440.43
z=4
1456.98
z=?
1471.68
z=?
1488.63
z=3
1499.99
z=3
Relative Abundance
m/z
MS/MS spectrum of m/z 1345.925³⁺
1186.5974
z=2
204.0862
z=1
366.1385
z=1
325.1120
z=1
487.3233
z=1
587.3034
z=?
791.0649
z=3
1085.0583
z=2
1056.5376
z=2
1288.1393
z=2
1450.1810
z=2
1612.7479
z=2
1901.9449
z=?
2068.9915
z=?
2170.0835
z=1
2372.1843
z=1

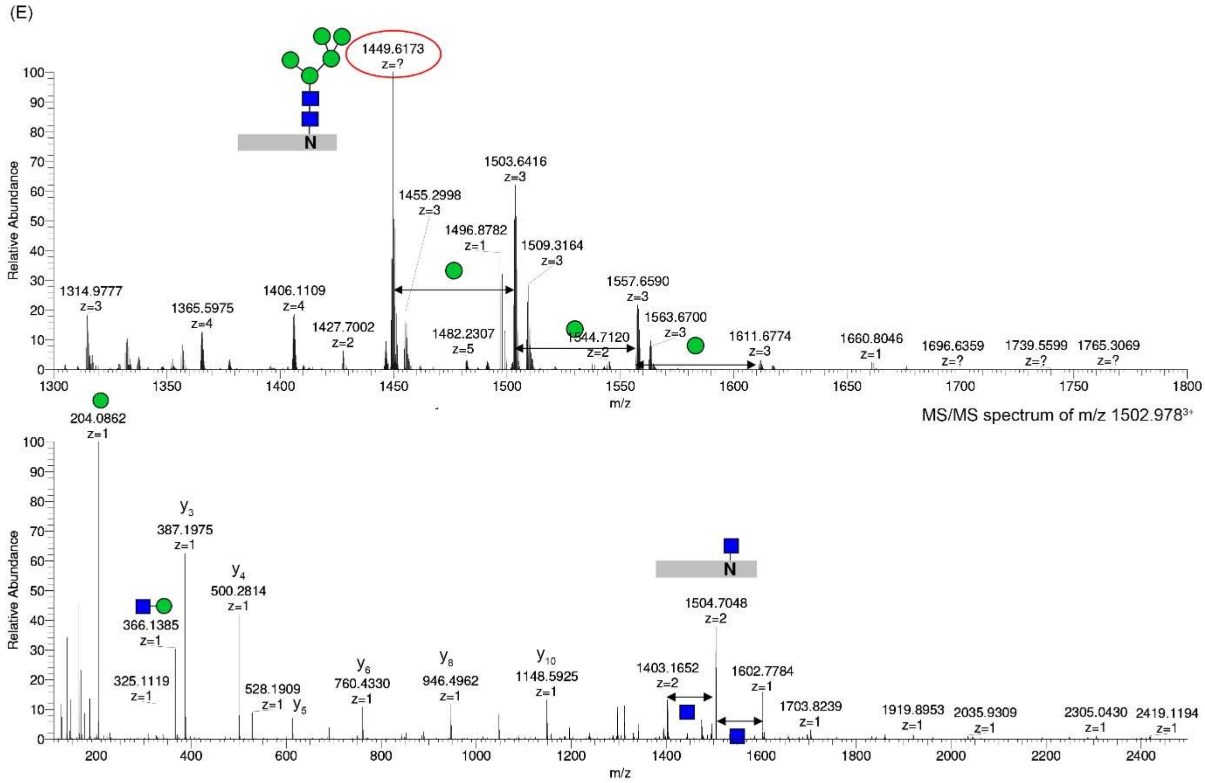

Supp. Figure 2 (to Figure 1D). Glycosylation profiling of the OST subunits.
The MS (upper panels) and MS/MS (lower panels) spectra from **(A)** N99 of Ost1p, **(B)** N217 of Ost1p, **(C)** N539 of Stt3p, **(D)** N60 of Wbp1p and **(E)** N332 of Wbp1p are shown. The m/z and structure details are summarized in Supp. Table 4. The m/z circled in red indicates the m/z corresponding to the drawn glycopeptide. Blue squares represent HexNAc; green circles represent mannose (a hexose).

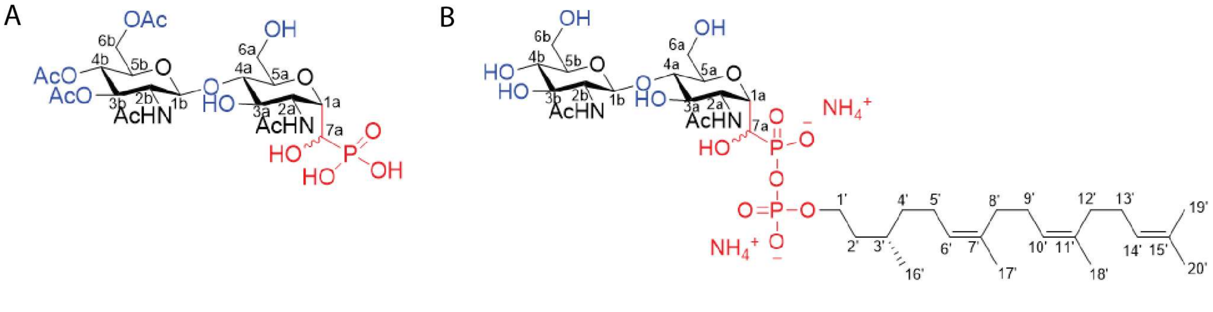

Supp. Figure 3. Structures of a synthesis precursor and the final compound Ia (non-hydrolyzable LLO)
(A) Chitobiose hydroxy-phosphonate (minor diastereomer): (((2S,3R,4R,5S,6R)-3-acetamido-5-(((2S,3R,4R,5S,6R)-3-acetamido-4,5-diacetoxy-6-(acetoxymethyl)tetrahydro-2H-pyran-2-yl)oxy)-4-hydroxy-6-(hydroxymethyl)tetrahydro-2H-pyran-2-yl)(hydroxy)methyl)phosphonic acid.
(B) Non-hydrolyzable LLO **Ia,** GlcNAc$_2$-(OH)CPP-(*S*)-CitronellylNeryl (C20) (minor diastereomer): (((2S,3R,4R,5S,6R)-3-acetamido-5-(((2S,3R,4R,5S,6R)-3-acetamido-4,5-dihydroxy-6-(hydroxymethyl)tetrahydro-2H-pyran-2-yl)oxy)-4-hydroxy-6-(hydroxymethyl)tetrahydro-2H-pyran-2-yl)(hydroxy)methyl)phosphonic ((S,6Z,10Z)-3,7,11,15-tetramethylhexadeca-6,10,14-trien-1-yl phosphoric) anhydride.
